# *KIAA0101* as a new diagnostic and prognostic marker, and its correlation with gene regulatory networks and immune infiltrates in lung adenocarcinoma

**DOI:** 10.18632/aging.104144

**Published:** 2020-11-20

**Authors:** Sheng Hu, Weibiao Zeng, Wenxiong Zhang, Jianjun Xu, Dongliang Yu, Jinhua Peng, Yiping Wei

**Affiliations:** 1 Department of Thoracic Surgery, The Second Affiliated Hospital of Nanchang University, Nanchang, China

**Keywords:** *KIAA0101/PCLAF*, lung adenocarcinoma, LUAD, biomarker, survival analysis

## Abstract

Proliferating cell nuclear antigen binding factor (encoded by *KIAA0101/* *PCLAF*) regulates DNA synthesis and cell cycle progression; however, whether the level of *KIAA0101* mRNA in lung adenocarcinoma is related to prognosis and tumor immune infiltration is unknown. In patients with lung adenocarcinoma, the differential expression of *KIAA0101* was analyzed using the Oncomine, GEPIA, and Ualcan databases. The prognosis of patients with different *KIAA0101* expression levels was evaluated using databases such as Prognostan and GEPIA. Tumor immune infiltration associated with *KIAA0101* was analyzed using TISIDB. Linkedmics was used to perform gene set enrichment analysis of *KIAA0101*. *KIAA0101* expression in lung adenocarcinoma tissues was higher than that in normal lung tissues. Patients with lung adenocarcinoma with low *KIAA0101* expression had a better prognosis than those with high *KIAA0101* expression. We constructed the gene regulatory network of *KIAA0101* in lung adenocarcinoma. *KIAA0101* appeared to play an important role in the regulation of tumor immune infiltration and targeted therapy in lung adenocarcinoma. Thus, *KIAA0101* mRNA levels correlated with the diagnosis, prognosis, immune infiltration, and targeted therapy in lung adenocarcinoma. These results provide new directions to develop diagnostic criteria, prognostic evaluation, immunotherapy, and targeted therapy for lung adenocarcinoma.

## INTRODUCTION

Lung cancer remains one of the most common cancers in men worldwide [1, 2]. In the United States, between 2008 and 2014, the 5-year relative survival rate for lung cancer was only 19% [2]. Lung cancer includes multiple subtypes, and the proportion of lung adenocarcinoma (LUAD) has increased in recent years. Despite significant improvements in chemotherapy and molecular targeted therapy, the survival rate of LUAD is still unsatisfactory. Tumor recurrence and metastasis are major problems in the clinical treatment of LUAD [3]. Drug resistance to common gene targeted drugs and therapeutic effect differences between different patients can occur [4]. Pabla, Conroy [5] found that many genes represented by *KIAA0101* are immune related genes of adenocarcinoma, and *KIAA0101* and other genes might affect the presentation of tumor immune antigens. However, the specific relationship between *KIAA0101* and immune escape is not clear. In this study, we found that the expression of *KIAA0101* correlated negatively with a proportion of tumor infiltrating lymphocytes, and the expression of *KIAA0101* was correlated negatively with MHC molecule expression. Therefore, we carried out a study on the relationship between the expression of *KIAA0101* and immune escape in lung adenocarcinoma, and further studied the effect of *KIAA0101* expression on immunotherapy of lung cancer. Our study showed that overexpression of *KIAA0101* promotes immune escape in lung adenocarcinoma. Therefore, searching for novel molecular biomarkers and improving immunotherapy of tumors in the diagnosis and treatment of LUAD are important [6].

KIAA0101 is a proliferating cell nuclear antigen (PCNA) binding factor discovered by Yu et al. in 2001 [7] using yeast two-hybrid experiments. It has different functions to other binding factors of PCNA, such as p21 and p57, in that KIAA0101 does not inhibit DNA replication and cell cycle process. PCNA-binding proteins act as regulators of DNA repair during DNA replication. Following DNA damage, they also facilitate the bypass of replication-fork-blocking lesions. KIAA0101 also acts as a regulator of centrosome number. However, the role of KIAA0101 in lung adenocarcinoma has not been determined. The protein encoded by *KIAA0101* contains eight domain chains (Supplementary Table 1) [8]. We noted that *KIAA0101* expression in lung adenocarcinoma was confirmed using an antibody in The Human Protein Atlas (Supplementary Figure 1) [9]. The expression of *KIAA0101* in the nucleus was significantly increased and slightly enhanced in the cytoplasm. The expression of *KIAA0101* increased in a variety of cancer tissues and cells, especially in liver cancer [10], breast cancer [11, 12], gastric cancer [13], and other cancers.

The present study aimed to explore the relationship between *KIAA0101* expression and the prognosis and immune infiltration of LUAD. We also searched for *KIAA0101*-related gene regulatory networks. *KIAA0101* expression was analyzed via the Oncomine, GEPIA, and Ualcan databases. The prognosis of LUAD related to *KIAA0101* was evaluated using prognostan and other databases. Analysis of tumor immune infiltration related to *KIAA0101* was analyzed using TISIDB. Gene set enrichment analysis was performed using Linkedmics. We hoped to provide new biomarkers related to *KIAA0101* for future targeted therapy of LUAD. In addition, we assessed whether we could predict the survival of patients with LUAD based on the expression of *KIAA0101*. The discovery of the gene regulatory network of *KIAA0101* will open new avenues for further studies on the epigenetics of *KIAA0101*. The study of immune infiltration related to *KIAA0101* will provide new directions for immunotherapy of lung adenocarcinoma.

## RESULTS

### Differential expression of *KIAA0101* in lung adenocarcinoma

**Figure 1:**
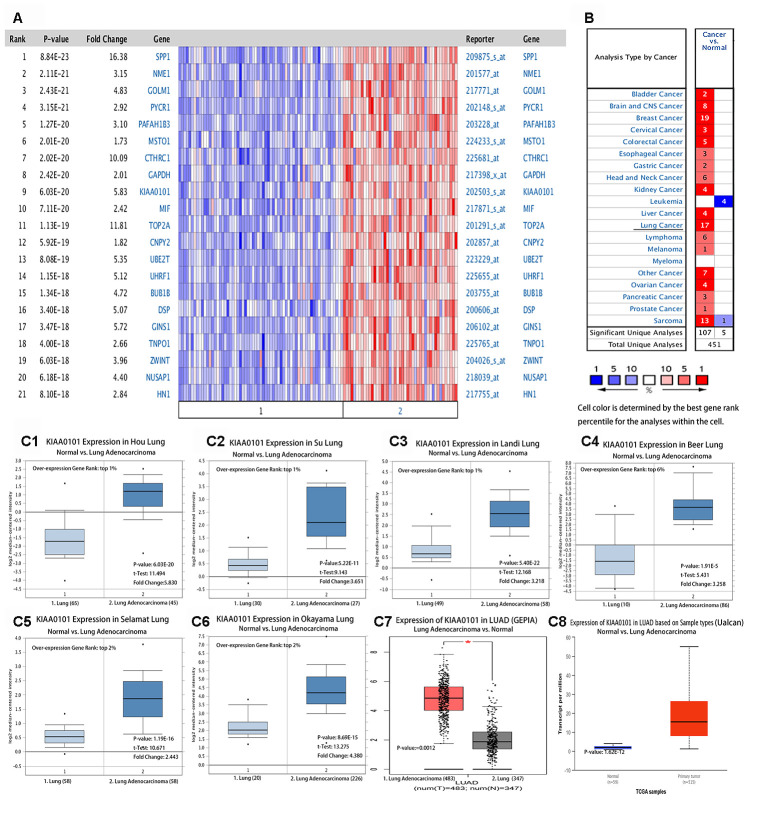
****(**A**) Multigene view of lung adenocarcinoma Heat Maps Comparison of all Genes in the study of Hou lung [50]. The expression of *KIAA0101* in lung adenocarcinoma was higher than that in the normal control group. (**B**) Summary view of *KIAA0101*. The transcription level of *KIAA0101* in different types of cancer. Parameter setting: gene: *KIAA0101*, threshold (P-value): 0.001, threshold (fold change):2, threshold (gene rank): 10%, data type: DNA and mRNA. Note: The color is standardized by the Z-score to describe the relative value in the row. They cannot be used to compare values between rows. Among them, Red signifies gene overexpression or copy gain in the analyses represented by that cell in the table; blue represents the gene's underexpression or copy loss in those analyses. Datasets comprised samples represented as microarray data measuring either mRNA expression on primary tumors, cell lines, or xenografts. (**C**) Transcription of *KIAA0101* in lung adenocarcinoma (from Oncomine, GEPIA, and Ualcan). mRNA expression levels of *KIAA0101* were significantly higher in lung adenocarcinoma than in normal tissue. (**C1**–**C6**) Shown are the fold change, associated p-values, and overexpression Gene Rank, based on Oncomine 4.5 analysis. Box plot showing *KIAA0101* mRNA levels in, respectively, the Hou Lung, Su Lung, Landi Lung, Beer Lung, Selamat Lung and Okayama Lung datasets [50]. (**C7**) Expression of *KIAA0101* in LUAD based on GEPIA analysis; the p-value was 0.0012. (**C8**) Shows the expression of *KIAA0101* in LUAD based on Ualcan analysis; the p-value was 1.62E-12.

Figure 1A shows a heat map of multiple genes that were overexpressed in LUAD. The top 23 ranked differentially expressed genes were obtained based on the log2 median centered ratio. After a genome-wide analysis, we identified these 23 genes, including *KIAA0101*, which were overexpressed in LUAD (Figure 1A, red text genes). We used Oncomine to analyze the expression distribution of *KIAA0101* mRNA in different tumors (Figure 1B). Oncomine collected these datasets from public repositories such as Gene Expression Omnibus (GEO) and Array Express. The results showed that when comparing cancer samples with normal samples, *KIAA010*1 was overexpressed in 17 LUAD datasets, but was not under-expressed in any of the LUAD datasets (Figure 1B). The thresholds were a P value less than 0.001, fold change greater than two, and gene rank in the top 10%. All results were statistically significant. The results were analyzed statistically using Student's t test. The mRNA expression level of *KIAA0101* was significantly higher in LUAD than in normal tissues. Figure 1C1–1C8 show the data from Oncomine, GEPIA, and Ualcan comparisons of *KIAA0101* mRNA levels between eight different gene sets of adenocarcinoma and normal tissues. All the results showed that the expression level of *KIAA0101* in LUAD tissues was significantly higher than that in normal lung tissues, as assessed using Student's t test. Further analysis of various clinical features of LUAD in the TCGA showed that *KIAA0101* was highly transcribed. In subgroup analysis based on stage, race, sex, age, smoking habit, and metastasis in the UALCAN database, the transcription level of *KIAA0101* in patients with LUAD was significantly higher than that in healthy people (Supplementary Figure 14A–14F). Therefore, the expression of *KIAA0101* could be used as a potential diagnostic marker for LUAD [14].

### *KIAA0101* mRNA levels predict prognosis in patients with lung adenocarcinoma

**Figure 2:**
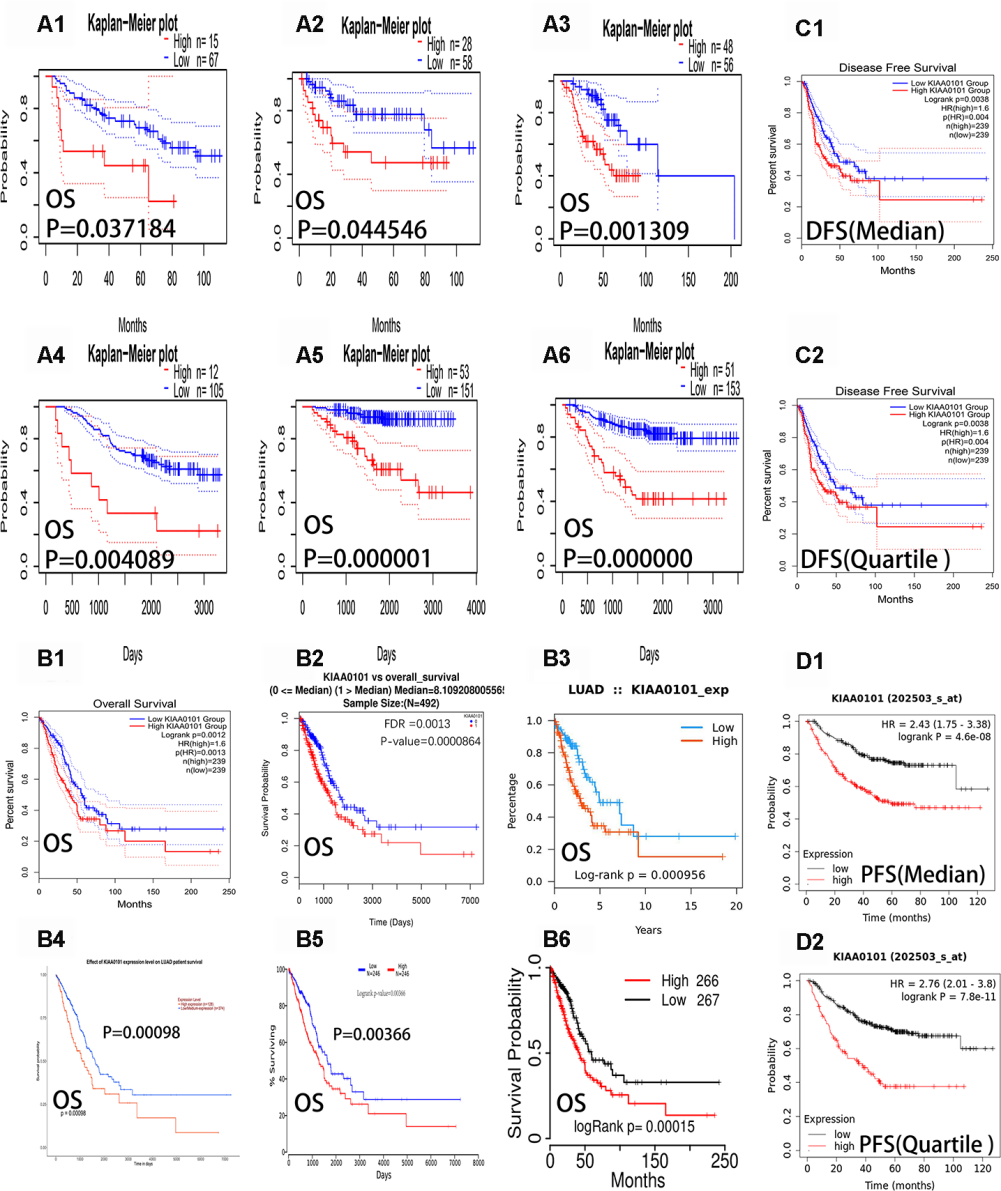
**Overall survival curves, progression-free survival curves and disease-free survival curves of *KIAA0101* in lung adenocarcinoma.** The blue curves represent patients with lung adenocarcinoma with low expression of *KIAA0101*, and the red curves represent patients with lung adenocarcinoma with high expression of *KIAA0101*. (**A1**–**A6**) Six survival curves representing the six different data sets in Table 1 (from PrognoScan databases), respectively. (**B1**–**B6**) The six overall survival curves from the GEPIA, Linkedmics, Ualcan, TISIDB, Oncolnc, and TCGA portal databases, respectively. (**C1**–**C2**) Disease free survival curves (DFS) of *KIAA0101* from the GEPIA database. (**D1–D2**) Progression free survival curves (PFS) of *KIAA0101* from Kaplan Meier-plotter.

Our next analysis showed that the expression of *KIAA0101* had a significant effect on the prognosis of LUAD (Table 1). Figure 2A1–2A6 show the overall survival curve of five datasets (Jacob-00182-CANDF, MICHIGAN-LC, Jacob-00182-MSK, GSE13213, GSE31210) and the relapse free survival curve of GSE31210 datasets. (Figure 2A1–2A6) show six survival curves representing the five different data sets in Table 1 (from the PrognoScan[15] database). We could observe the blue curves (low expression) were higher than the red curves (high expression) in the Kaplan-Meier plots (Figure 2A1–2A6). In addition, we could see from Table 1 that all six Hazard Ratios (HR) were greater than 1.4, the HR [95% confidence interval (CI)-low CI-up] range was greater than one. The Cox p-values were less than 0.05, indicating statistical significance. The results showed that low expression of *KIAA0101* was associated with a good prognosis in patients with LUAD. Figure 2B1–2B6 show the overall survival curves of patients using data from the GEPIA [16], LinkedOmics [17], Ualcan [14], TISIDB [18], OncoLnc [19], and TCGA portal [20] databases. We found that patients with LUAD with low *KIAA0101* expression had a better prognosis than those with high *KIAA0101* expression in six the overall survival (OS) curves (P-value < 0.01). Figure 2C1 and 2C2 show the disease free survival curves (DFS) of patients using data from the GEPIA database. Figure 2D1 and 2D2 show the progression free survival curves (PFS) of patients using data from Kaplan Meier-plotter. We found that patients with LUAD with low *KIAA0101* expression had a better prognosis than those with high *KIAA0101* expression in four survival curves (P-value < 0.01). These curves reflect the fact that high expression of *KIAA0101* is associated negatively with the prognosis of patients with LUAD.

**Table 1:** Survival analysis of *KIAA0101* mRNA in lung adenocarcinoma patients (From PrognoScan).

	**DATASET**	**PROBE ID**	**ENDPOINT**	**Number**	**Ln (HR high/HR low)**	**COXP-VALUE**	**Ln (HR)**	**HR [95% CI-low CI-up]**
A1	Jacob-00182-CANDF	202503_s_at	Overall Survival	82	1.03	0.037184	0.57	1.76 [1.03 - 2.99]
A2	MICHIGAN-LC	D14657_at	Overall Survival	86	0.96	0.044546	0.43	1.54 [1.01 - 2.35]
A3	Jacob-00182-MSK	202503_s_at	Overall Survival	104	1.12	0.001309	0.7	2.01 [1.31 - 3.07]
A4	GSE13213	A_23_P117852	Overall Survival	117	1.24	0.004089	0.36	1.44 [1.12 - 1.84]
A5	GSE31210	202503_s_at	Overall Survival	204	1.93	0.000001	1.29	3.65 [2.16 - 6.17]
A6	GSE31210	202503_s_at	Relapse Free Survival	204	1.5	0	1	2.73 [1.90 - 3.91]

### Gene set enrichment analysis of *KIAA0101* functional networks

### Gene set enrichment analysis (GSEA) of KIAA0101 functional networks

**Figure 3:**
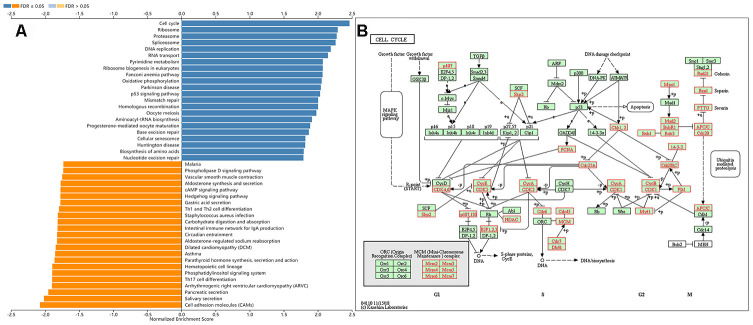
**Gene set enrichment analysis of the *KIAA0101* via the KEGG pathway.** (**A**) Bar chart for gene set enrichment analysis of the *KIAA0101* via the KEGG pathway. (**B**) KEGG pathway annotations of the cell cycle pathway (hsa04110). Red denotes leading edge genes; green denotes the remaining genes.

(using LinkedOmics [17]). Table 2 shows the top five most significant of the seven GSEA results. All the results were statistically significant; the normalized enrichment scores (NES) were all greater than one; the P-values were all less than 0.05, and the false discovery rates (FDR) were all less than 0.05. Figure 3A shows a bar graph of the Kyoto Encyclopedia of Genes and Genomes (KEGG) enrichment analysis. Supplementary Figure 2A–2P display the enrichment plot of pathways in the enrichment results from Figure 3A, with the top 8 and the bottom 8 according to the normalized enrichment score. We observed that the top five pathways of the KEGG [21] enrichment analysis were the cell cycle (Figure 3B), ribosome (Supplementary Figure 3), proteasome (Supplementary Figure 4), spliceosome (Supplementary Figure 5), and DNA replication (Supplementary Figure 6). The gene ontology (GO) results for the gene sets (Cellular Component) included condensed chromosome, chromosomal region, mitochondrial protein complex, ribosome, and spindle. The GO results for the gene sets (Biological Process) included chromosome segregation, DNA replication, cell cycle checkpoint, double-strand break repair, and spindle organization. The GO results for the gene sets (Molecular Function) included structural constituent of ribosome, catalytic activity, acting on DNA, single-stranded DNA binding, helicase activity and catalytic activity, and acting on RNA. Kinase targets included CDK1 (cyclin-dependent kinase 1), PLK1 (polo-like kinase 1), AURKB (aurora kinase B), CDK2 (cyclin-dependent kinase 2), and ATR (ATR serine/threonine kinase). MicroRNA targets included MIR-484, MIR-512-3P, MIR-19A, MIR-19B, MIR-219, and MIR-326. Transcription factor targets included E2F Q6, E2F Q4, E2F4DP1 01, E2F1 Q6, and E2F 02. Supplementary Tables 2–8 show the details of all Leading Edge Genes specifically contained in each gene set.

**Table 2:** Gene Ontology (GO) (Cellular Component), GO (Biological Process), GO (Molecular Function), Kyoto Encyclopedia of Genes and Genomes (KEGG pathway), Kinase, miRNA, and transcription factor-target networks of *KIAA0101* in lung adenocarcinoma (LinkedOmics).

**Enriched Category**	**Geneset**	**LeadingEdgeNum**	**NES**	**P Value**	**FDR**
**GO (Cellular Component)**	condensed chromosome	66	2.6425	0	0
	chromosomal region	94	2.5825	0	0
	mitochondrial protein complex	152	2.3976	0	0
	ribosome	143	2.3858	0	0
	spindle	70	2.3837	0	0
**GO (Biological_Process)**	chromosome segregation	97	2.6361	0	0
	DNA replication	96	2.4830	0	0
	cell cycle checkpoint	60	2.3720	0	0
	double-strand break repair	61	2.3053	0	0
	spindle organization	44	2.2998	0	0
**GO (Molecular_Function)**	structural constituent of ribosome	109	2.3582	0	0
	catalytic activity, acting on DNA	69	2.2380	0	0
	single-stranded DNA binding	43	2.1594	0	0
	helicase activity	43	2.0895	0	0
	catalytic activity, acting on RNA	122	2.0460	0	0
**KEGG pathway**	Cell cycle	48	2.4639	0	0
	Ribosome	100	2.2904	0	0
	Proteasome	40	2.2761	0	0
	Spliceosome	63	2.2601	0	0
	DNA replication	27	2.1887	0	0
**Kinase Target**	Kinase_CDK1	74	2.3361	0	0
	Kinase_PLK1	32	2.3154	0	0
	Kinase_AURKB	35	2.1660	0	0
	Kinase_CDK2	73	2.1657	0	0
	Kinase_ATR	20	2.0601	0	0
**miRNA Target**	GAGCCTG,MIR-484	40	-(1.7728	0	0.025589
	CAGCACT,MIR-512-3P	53	(1.6953	0	0.029684
	TTTGCAC,MIR-19A,MIR-19B	148	(1.7059	0	0.034546
	GACAATC,MIR-219	60	(1.7191	0	0.039237
	CCCAGAG,MIR-326	58	(1.6503	0	0.039481
**Transcription Factor Target**	V$E2F_Q6	87	2.2583	0	0
	V$E2F_Q4	86	2.2538	0	0
	V$E2F4DP1_01	94	2.2226	0	0
	V$E2F1_Q6	96	2.2207	0	0
	V$E2F_02	93	2.1958	0	0

Abbreviations: Leading EdgeNum, the number of leading edge genes; FDR, false discovery rate from Benjamini and Hochberg from gene set enrichment analysis (GSEA); NES, Normalized Enrichment Score. It is generally considered that the absolute value of NES ≥ 1.0, NOM p-val ≤ 0.05, FDR q-val ≤ 0.25 are significant gene sets. The annotation was found in the Molecular Signatures Database (MSigDB) for transcription factors (TF).

### Pearson correlated genes, miRNAs and lncRNAs of *KIAA0101*

**The differentially expressed genes correlate highly with KIAA0101 in lung adenocarcinoma (LinkedOmics)**

**Figure 4:**
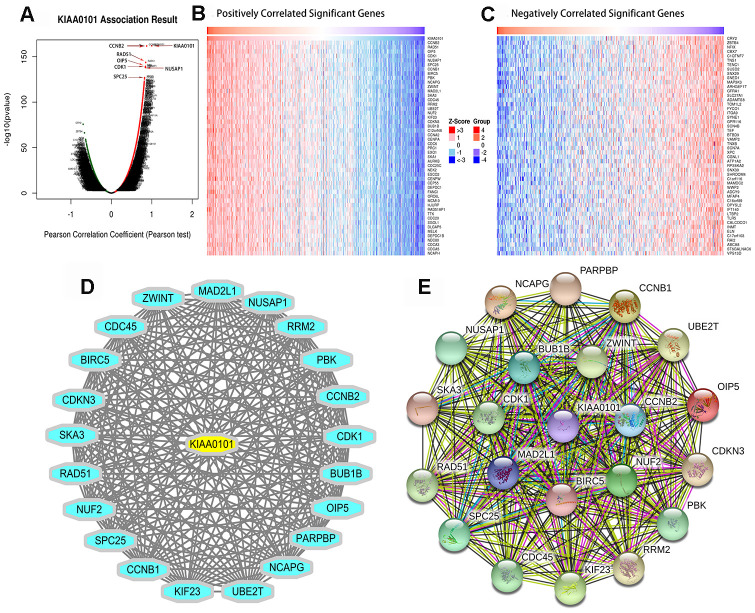
***KIAA0101* highly correlated genes.** (**A**) Pearson test was used to analyze the correlation between *KIAA0101* and differentially expressed genes in LUAD. (**B**, **C**) Heat maps showing the genes that correlated positively and negatively with *KIAA0101* in LUAD (top 50). Red indicates positively correlated genes, green indicates negatively correlated genes. (**D**, **E**) The network view summarizes the predicted association network of proteins that are strongly related to the protein product of *KIAA0101*. Network nodes are proteins. The edge represents the functional association of the prediction. A red line indicates the existence of fusion evidence, a green line represents neighborhood evidence, a blue line represents co-occurrence evidence, a purple line represents experimental evidence, a yellow line represents text mining evidence, and a light blue line represents database evidence.

Figure 4A shows a Volcano plot of the results of the Pearson correlation test of *KIAA0101* in LUAD. The red and green dots in Figure 4A represent the differentially expressed genes associated with *KIAA0101*. Figure 4B and 4C show heat maps of the top 50 genes with strong positive and negative correlations with *KIAA0101* in LUAD. Figure 4D and 4E show the network views summarizing the predicted association network of proteins strongly related to *KIAA0101*. Supplementary Figure 7A–7P show scatter diagrams of *KIAA0101* and the first 16 genes that are highly related to *KIAA0101*. From these figures, we observed that the P-value was less than 1E-118; the FDR (BH) was less than 1E-113; and the Pearson correlation coefficient was > 0.8 (Figure 7A–7H), the Pearson correlation coefficient was <-0.6 (Figure 7I–7P). All the genes in the map had a strong positive correlation with *KIAA0101*.

**miRNAs associated with KIAA0101 in patients with LUAD and their prognostic significance**

**Figure 5:**
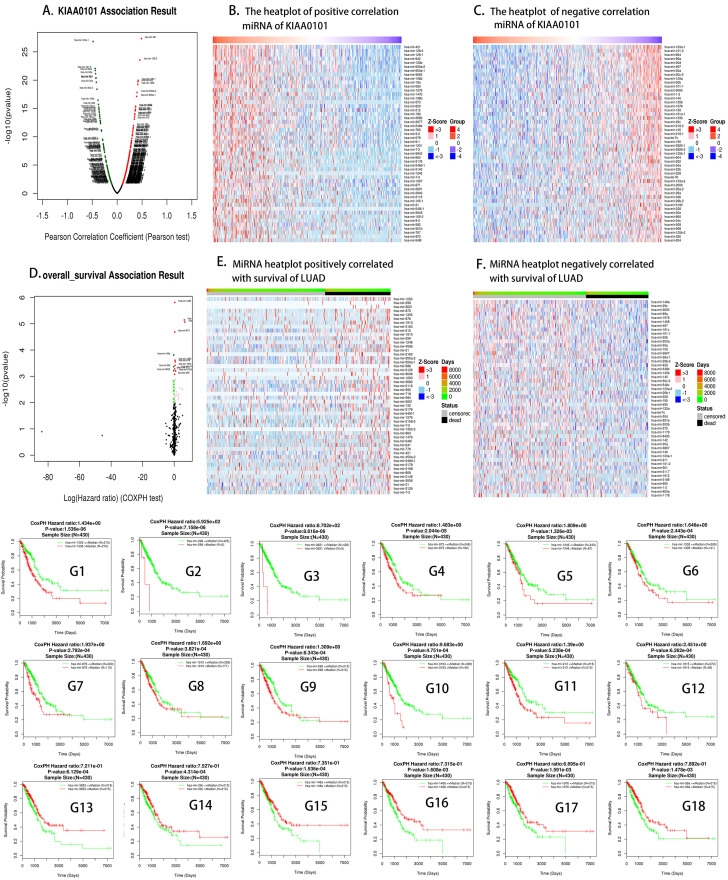
***KIAA0101* highly correlated miRNAs.** (**A**) Volcano plot of *KIAA0101* related miRNAs; (**B**, **C**) positively and negatively correlated significant miRNA heat plots of *KIAA0101*. (**D**) The miRNA volcano map related to the overall survival of LUAD; (**E**, **F**) miRNAs positively and negatively related to the overall survival of LUAD, respectively; (**G1**–**G18**) the survival curves of *KIAA0101* related miRNAs; green represents low expression of the corresponding miRNA, while red represents high expression of the corresponding miRNA.

We performed an miRNA Pearson correlation test and a COXPH test of *KIAA0101* (Figure 5). From the Volcano plot of *KIAA0101*-related miRNAs (Figure 5A), we found that the miRNAs hsa-mir-421, hsa-mir-133a-1, hsa-mir-128-2, hsa-mir-101-2, and hsa-mir-664 correlated strongly with *KIAA0101*. We identified the positive and negative miRNAs of *KIAA0101* in LUAD (top 50) from the miRNA heat plots of *KIAA0101* (Figure 5B, 5C). Supplementary Figure 8 shows the scatter plots of positive (Supplementary Figure 8A1–8A10) and negative (Supplementary Figure 8B1–8B10) correlations between the expression of *KIAA0101* and miRNAs. Their P-values were less than 1E-14, and their FDRs (BH) were less than 1E -12, thus the results were statistically significant. We constructed a Volcano plot of *KIAA0101* correlated miRNAs with significance for patient survival in lung adenocarcinoma (Figure 5D). One hundred miRNAs that correlated positively and negatively with the prognosis of patients with LUAD were identified from Figure 5E and 5F. Figure 5G1–5G12 show the *KIAA0101*-related miRNAs with low expression that were associated with good prognosis. Figure 5G13–5G18 show the *KIAA0101*-related miRNAs with high expression that were associated with good prognosis. The P values were less than 0.001 and the COXPH hazard ratios were greater than one, indicating that the results were statistically significant.

**Long noncoding RNAs (lncRNAs) that correlated with KIAA0101 and were significant for patient survival**

**Figure 6:**
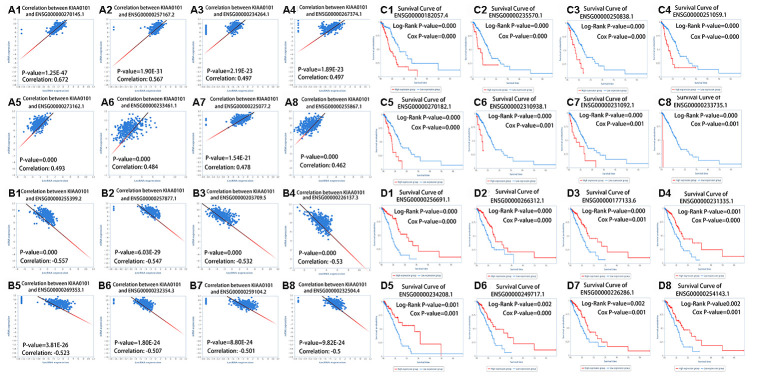
***KIAA0101* highly correlated lncRNAs.** (**A1**–**A8**) Scatter plots of eight kinds of lncRNAs that correlated positively with *KIAA0101* expression. (**B1**–**B8**) Scatter plots of eight kinds of lncRNAs that correlated negatively with *KIAA0101* expression. (**C1**–**C8**) Survival curves of eight kinds of lncRNAs; patients with low expression of lncRNAs have a higher survival rate. (**D1**–**D8**) Survival curves of eight kinds of lncRNAs; patients with high expression of lncRNAs have a higher survival rate.

Figure 6 shows scatter plots of the lncRNAs that correlated positively (Figure 6A1–6A8) and negatively (Figure 6B1–6B8) with *KIAA0101* expression. The results were statistically significant. Figure 6C1–6C8 show the OS curves of lncRNAs related to *KIAA*0101 expression, in which the Cox P-value of all lncRNAs were less than or equal to 0.001 and the log-rank P-value of all lncRNAs were less than or equal to 0.002. Figure 6C1–6C8 show the *KIAA0101*-related lncRNAs with low expression that were associated with better prognosis of patients with LUAD. Figure 6D1–6D8 show the *KIAA0101*-related lncRNAs with low expression that were associated with better prognosis of patients with LUAD.

### The relationship between immune infiltration and the expression of *KIAA0101* in lung adenocarcinoma

**Figure 7:**
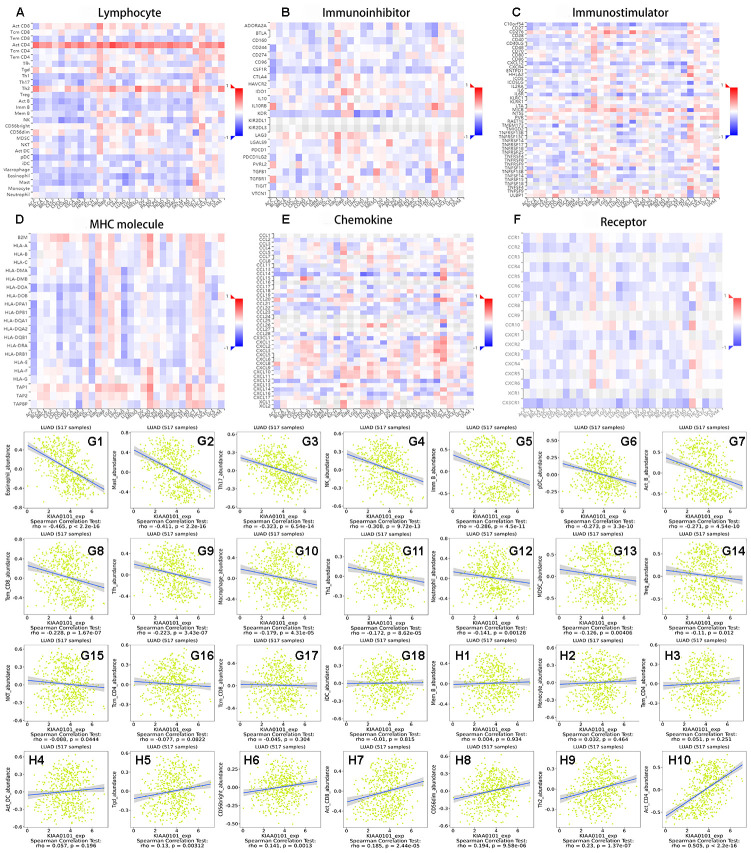
**The relationship between immune infiltration and expression of *KIAA0101* in lung adenocarcinoma.** (**A**–**F**) Heat maps of *KIAA0101* expression and lymphocytes, immunoinhibitors, immunostimulators, MHC molecules, chemokines, and receptors in different cancers. (**G1**–**G18**) are scatter plots of the negative correlation between *KIAA0101* expression and lymphocytes in the treatment of lung adenocarcinoma. (**H1**–**H10**) are scatter plots of the positive correlation between *KIAA0101* expression and lymphocytes in the treatment of lung adenocarcinoma.

Figure 7A–7F show heat maps of the correlation between the expression of *KIAA0101* in multiple cancers and different tumor infiltrating lymphocytes (TILs), immunoinhibitors, immunostimulators, MHC molecules, chemokines, and receptors. Figure 7G1–7G18 show scatter diagrams of tumor infiltrating lymphocytes that correlated negatively with *KIAA0101* expression in LUAD. Figure 7H1–7H10 show scatter diagrams of TILs that correlated positively with *KIAA0101* expression in LUAD. When the expression of *KIAA0101* increased, the number of activated CD4 T cells increased. The numbers of Th1 cell, Th17 cells, and follicular helper cell were negatively related to the expression of *KIAA0101*, and Th2 cell numbers were positively related to the expression of *KIAA0101* (P < 0.05). *KIAA0101* expression correlated positively with the numbers of effector memory CD8 T cells, natural killer cells, regulatory T cells, activated B cells, immature B cells, plasmacytoid dendritic cells, macrophages, eosinophil, mast cells, and neutrophils (all P < 0.05). Supplementary Figures 9–13 show scatter plots of *KIAA0101* expression related to immunoinhibitors, immunostimulators, MHC molecules, chemokines, and chemokine receptors, respectively. Most chemokine receptors correlated negatively with the expression of *KIAA0101* (Supplementary Figure 13). The majority of MHC molecules correlated negatively with the expression of *KIAA0101* (Supplementary Figure 11).

We found that the metabolism of seven drugs upregulated the expression of *KIAA0101* (Table 3). They included Acetaminophen [22], Estradiol [23], Genistein [23], Progesterone [24], Resveratrol [25], Rifampicin [26], and Valproic acid [27]. The metabolism of 24 drugs downregulated the expression of *KIAA0101*. They included amoxicifene, estradiol, yolk, copper, and cyclosporine (Table 3). The metabolism of Estradiol [23, 28], Genistein [23, 29], and Progesterone [24, 30] could both upregulate and downregulate the expression of *KIAA0101* (Table 3).

**Table 3:** The Pharmaco-transcriptomics of *KIAA0101* (Up or downregulation of genes caused by the metabolism of pharmaceutical compounds).

**DRUG**	**CHANGE**	**INTERACTION**	**REFERENCES (PMID)**
Acetaminophen	upregulated	Acetaminophen results in increased expression of *KIAA0101* mRNA	25704631
Afimoxifene	downregulated	Afimoxifene results in decreased expression of *KIAA0101* mRNA	16514628
Estradiol	downregulated	Estradiol results in decreased expression of *KIAA0101* mRNA	23019147
Estradiol	upregulated	Estradiol results in increased expression of *KIAA0101* mRNA	16474171
Custirsen	downregulated	CC-8490 results in decreased expression of *KIAA0101* mRNA	15604281
Copper	downregulated	Binding with copper resulted in decreased expression of *KIAA0101* mRNA	20971185
Cyclosporine	downregulated	Cyclosporine results in decreased expression of *KIAA0101* mRNA	20106945
Dasatinib	downregulated	Dasatinib results in decreased expression of *KIAA0101* mRNA	20579391
Dronabinol	downregulated	Dronabinol results in decreased expression of *KIAA0101* mRNA	18454173
Calcitriol	downregulated	Calcitriol results in decreased expression of *KIAA0101* mRNA	21592394
Fluorouracil	downregulated	Fluorouracil results in decreased expression of *KIAA0101* mRNA	17039268
Fulvestrant	downregulated	fulvestrant results in decreased expression of *KIAA0101* mRNA	16514628
Genistein	downregulated	Genistein results in decreased expression of *KIAA0101* mRNA	19371625
Genistein	upregulated	Genistein results in increased expression of *KIAA0101* mRNA	16474171
Lucanthone	downregulated	Lucanthone results in decreased expression of *KIAA0101* mRNA	21148553
Metamfetamine	downregulated	Methamphetamine results in decreased expression of *KIAA0101* mRNA	25290377
Methotrexate	downregulated	Methotrexate results in decreased expression of *KIAA0101* mRNA	24657277
Palbociclib	downregulated	Palbociclib results in decreased expression of *KIAA0101* mRNA	22869556
Phenethyl Isothiocyanate	downregulated	Phenethyl isothiocyanate results in decreased expression of *KIAA0101* mRNA	26678675
Piroxicam	downregulated	Piroxicam results in decreased expression of *KIAA0101* mRNA	21858171
Progesterone	downregulated	Progesterone results in decreased expression of *KIAA0101* mRNA	21795739
Progesterone	upregulated	Progesterone results in increased expression of *KIAA0101* mRNA	18070364
Raloxifene	downregulated	Raloxifene Hydrochloride results in decreased expression of *KIAA0101* mRNA	16514628
Resveratrol	upregulated	Resveratrol results in increased expression of *KIAA0101* mRNA	19167446
Rifampicin	upregulated	Rifampin results in increased expression of *KIAA0101* mRNA	24552687
Silicon dioxide	downregulated	Silicon Dioxide analog results in decreased *expression* of KIAA0101 mRNA	25895662
Tamoxifen	downregulated	Tamoxifen results in decreased expression of *KIAA0101* mRNA	15604281
Testosterone	downregulated	Testosterone results in decreased expression of *KIAA0101* mRNA	21592394
Valproic acid	upregulated	Valproic Acid results in increased expression of *KIAA0101* mRNA	23179753
Vitamin E	downregulated	Vitamin E results in decreased expression of *KIAA0101* mRNA	19244175
Zoledronic acid	downregulated	Zoledronic acid results in decreased expression of *KIAA0101* mRNA	24714768

## DISCUSSION AND CONCLUSIONS

Early detection of LUAD is a challenge for clinicians. Low dose computed tomography (LDCT) has the advantages of simplicity and high sensitivity. Compared with the traditional chest film and tumor markers, the false negative rate of LDCT was reduced [31]. However, more accurate and timely biomarkers are needed to support a diagnosis of LUAD. It has been reported that *KIAA0101* is differentially expressed in ovarian cancer [32], adrenal cancer [33], renal cell carcinoma [10], hepatocellular carcinoma [10], and breast cancer [12]. However, there are few studies about *KIAA0101* in LUAD [34]. The results of the present study showed that *KIAA0101* was significantly differentially expressed in all lung adenocarcinomas (Figure 1). This is very interesting; however, it requires verification in further comparative studies.

We further explored the mechanism by which *KIAA0101* can act as a marker of LUAD. Hosokawa et al. [35] found that if *KIAA0101* was knocked down using small interfering RNA (siRNA), the DNA replication rate was greatly reduced in pancreatic cancer cell lines. The exogenous overexpression of *KIAA0101* enhanced the growth of cancer cells, suggesting the carcinogenicity of *KIAA0101*. They also found that inhibition of the *KIAA0101-PCNA* interaction significantly inhibited the growth of cancer cells. Lv et al. [11] found that knockdown of *KIAA0101* could promote the formation of p53 / SP1 complexes in breast cancer, and inhibited the proliferation and cell cycle process of breast cancer cells. Many studies have shown that *KIAA0101* is involved in the regulation of DNA replication and repair, cell cycle progression, and cell proliferation, and can reduce the apoptosis induced by UV [35–37]. Our results are consistent with these previous reports. KEGG enrichment analysis of *KIAA0101* identified the cell cycle, ribosome, proteasome, spliceosome, and DNA replication pathways as enriched. In LUAD, *KIAA0101* was related to kinase networks including CDK1, PLK1, AURKB, CDK2, and ATR. These kinases regulate cell cycle stability, chromosome separation, and the cell cycle during mitosis and meiosis [38, 39]. In fact, ATR is one of the key kinase regulators for genomic stability; it directly phosphorylates a variety of important substrates, including p53 protein and cyclin [40]. ATR kinase inhibitors can reduce the growth of tumor cells [41]. In addition, differentially expressed genes highly related to *KIAA0101* in lung adenocarcinoma were associated with the cell cycle, DNA replication and modification, mitosis, and meiosis. Thus, *KIAA0101* might regulate cell cycle progression and DNA replication and repair via the above kinases in LUAD [42]. Cell cycle progression and DNA replication occur mainly in the nucleus, which might also be the main reason for the markedly increased expression of *KIAA0101* in the nucleus and slightly enhanced expression in the cytoplasm.

Based on the above results (Figure 2), we found that the prognosis of patients with LUAD could be predicted by the expression of *KIAA0101*. We found that some drugs [43] could reduce the expression of *KIAA0101* (Table 3). The decrease in gene expression by these drugs [22] might be transient or concentration-dependent, and we will further evaluate the effect of different concentrations and times on *KIAA0101* expression in the future. Some drugs that upregulated the expression of *KIAA0101* might be potential carcinogens of LUAD. We also identified miRNAs and lncRNAs that are related to the expression of *KIAA0101* (Figures 5 and 6). The expression of these miRNAs and lncRNAs was related to the survival of patients with lung adenocarcinoma. Thus, these miRNAs and lncRNAs could also be used as potential biomarkers of lung adenocarcinoma [17, 44]. These miRNAs and lncRNAs could be further studied to improve the epigenetics of *KIAA0101* in lung adenocarcinoma.

The interaction between tumor cells and their surrounding matrix not only affects the occurrence and development of the disease, but also is closely related to the prognosis of patients [45]. Evidence suggests that TILs infiltrate tumor tissue and play a role in the disease by regulating anti-tumor immunity [46]. With the increase in *KIAA0101* expression, the numbers of Th1 cells, Th17 cells, and follicular helper cells decreased, and the number of Th2 cells increased. Type 1 T helper cells are positive immunoregulatory cells, and type 2 T helper cells are negative immunoregulatory cells. Thus, variation in *KIAA0101* expression leads to a shift of the Th1/Th2 ratio. The immunosuppressive state will seriously affect the anti-tumor immunity of the body, and eventually lead to the occurrence and development of tumors [47]. With the increase in *KIAA0101* expression, the number of effector memory CD8 T cells, natural killer cells, regulatory T cells, activated B cells, immature B cells, plasmacytoid dendritic cells, macrophages, eosinophil, mast cells, and neutrophils will decrease. This finding confirms the view that increased expression of *KIAA0101* reduces survival in patients with LUAD. Chemokine receptors are seven transmembrane G protein-coupled receptors. Their main function is to receive chemokine signals and further conduct signals, which plays an important positive role in promoting tumor peripheral immune infiltration [48]. The main function of MHC molecules is to participate in antigen presentation. MHC molecules play an important role in the differentiation and maturation of T cells, and play an important positive role in tumor peripheral immune infiltration [49]. When the expression of *KIAA0101* increased, the levels of most chemokine receptors and MHC molecules in patients decreased (Supplementary Figures 11, 12), which would further reduce the tumor peripheral immune function. This finding again confirmed that an increase of *KIAA0101* expression reduces the survival rate of patients with LUAD. Therefore, our research will contribute to the improvement of immunotherapy of LUAD and the development of new immunotherapy targets.

In conclusion, we speculated that *KIAA0101* is a new LUAD marker. The prognosis of patients with LUAD with high expression of *KIAA0101* was poor. *KIAA0101* is related to the cell cycle, ribosome, protocol, splicing, and DNA replication. The results showing the immune cell associations of *KIAA0101* could lead to new methods of immunotherapy of LUAD. The study of drug transcription and metabolism associated with *KIAA0101* might provide alternative drugs for the targeted treatment of LUAD. This study will provide new directions for the diagnosis, prognostic evaluation, immunotherapy, and targeted therapy of LUAD.

We must acknowledge the potential limitations of our analysis. Further experimental verification is needed. In addition, our study is limited to lung adenocarcinoma, and there was no analysis in other types of lung cancer. We hope that our next study will assess the association of *KIAA0101* with other types of lung cancer besides adenocarcinoma, to further compare the specific role of *KIAA0101* expression in the diagnosis and treatment of different types of lung cancer.

## MATERIALS AND METHODS

### Oncomine, GEPIA, and Ualcan analysis

All genes in the study of Hou Lung were analyzed and compared using Oncomine [50]. Overexpression of the genes in lung adenocarcinoma was compared with that in normal control tissue (log2 median-centered intensity). We used Oncomine to analyze the expression of *KIAA0101* in different tumor studies. In the Oncomine dataset, the naming convention lists the primary author's last name first, followed by the tissue type used in the study. We then used GEPIA [16] to analyze the expression of *KIAA0101* in a variety of tumors compared with that in normal tissue (Figure 1C). *KIAA0101* transcription in subgroups of patients with lung adenocarcinoma, stratified based on sex, age, and other criteria, were analyzed using the Ualcan database [14]. We also analyzed the differential expression of *KIAA0101* in LUAD tissues and normal lung tissues using Ualcan.

### PrognoScan, OncoLnc, and TCGA portal analysis

The prognosis of patients with LUAD with different expression levels of *KIAA0101* was evaluated using PrognoScan [15]. PrognoScan is a new database for the meta-analysis of the prognostic value of genes. We used GEPIA [16], LinkedOmics [17], Ualcan [14], TISIDB [18], OncoLnc [19], and TCGA portals [20] to construct the OS curves for patients with LUAD. We used GEPIA [16] to construct the DFS curves for patients with LUAD. We used Kaplan Meier-plotter to construct the PFS curves for patients with LUAD. The survival curves between patients with low expression of *KIAA0101* and patients with high expression of *KIAA0101* in lung adenocarcinoma were compared.

### LinkedOmics and TANRIC analysis

We conducted Gene Set Enrichment Analysis (GSEA) of KEGG pathway data [51], gene ontology cellular component, gene ontology biological process, gene ontology molecular function, a kinase target network, an miRNA target network, and a transcription factor target network of *KIAA0101*. We performed an miRNA Pearson correlation test and COXPH test of *KIAA0101* using Linkedomics [17]. We found the differentially expressed genes related to KIAA0101 in LUAD. We analyzed lncRNAs associated with *KIAA0101* using TANRIC [44] and assessed whether the level of lncRNA expression associated with *KIAA0101* had different impacts on the survival of patients.

### TISIDB analysis and DrugBank analysis

We used TISIDB [18] to analyze the relationship between the expression of *KIAA0101* and immune infiltration in LUAD. Transcriptome and clinical data of 30 cancer types from the Cancer Genome Atlas (TCGA) were assessed [18]. High throughput screening data were then used to detect the resistance and sensitivity of tumor cells to T-cell-mediated killing. We used Drugbank to analyze the pharmacotranscription of *KIAA0101* [52]. The DrugBank database is a unique bioinformatics and cheminformatics resource that combines detailed drug data with comprehensive drug target information.

## Supplementary Material

Supplementary Figures

Supplementary Tables
