# Supplementary material for: KIAA0101 as a new diagnostic and prognostic marker, and its correlation with gene regulatory networks and immune infiltrates in lung adenocarcinoma
Source: Aging (Albany NY). 2020 Nov 20;13(1):301–39. doi: 10.18632/aging.104144 (PMC7835026; doi:10.18632/aging.104144)
Supplement: Supplementary Figures [file aging-13-104144-s001.pdf]

SUPPLEMENTARY TABLES

Supplementary Table 1. Details of the eight domain chains of KIAA0101.

Serial number	ID	POSITION	IDENTITY			MAX IDENTITY	RESOLUTION	IN COMP-LEX WITH	EXPERIMENT TYPE	INTERFACE CAVITY
			START	STOP	SCORE					
A	4D2G_D	52 - 69	52	69	100	100	2.65 Å	PCNA	X-RAY DIFFRACTION	√
B	4D2G_E	52 - 69	52	69	100	100	2.65 Å	PCNA	X-RAY DIFFRACTION	√
C	6EHT_D	52 - 71	52	71	100	100	3.2Å	PCNA	X-RAY DIFFRACTION	√
D	6EHT_E	52 - 71	52	71	100	100	3.2Å	PCNA	X-RAY DIFFRACTION	√
E	6GWS_D	41-72	41	72	100	100	3.2Å	PCNA	X-RAY DIFFRACTION	√
F	6GWS_E	41-72	41	72	100	100	2.9Å	PCNA	X-RAY DIFFRACTION	√
G	6GWS_F	41-72	41	72	100	100	2.9Å	PCNA	X-RAY DIFFRACTION	√
H	6IIW_B	2-11	2	11	100	100	1.699Å	UHRF1	X-RAY DIFFRACTION	√

Supplementary Table 2. Significantly enriched gene ontology (GO) annotations (cellular components) of KIAA0101 in lung adenocarcinoma (LinkedOmics).

Description	Leading EdgeNum	FDR	Leading Edge Gene
condensed chromosome	66	0	RAD51, SPC25, CCNB1, BIRC5, NCAPG, ZWINT, MAD2L1, SKA3, NUF2, BUB1B, CENPA, SKA1, AURKB, NEK2, CENPW, HJURP, NDC80, CDCA5, NCAPH, BUB1, ZWILCH, CENPK, KIF2C, AURKA, CENPN, TOP2A, CENPM, PLK1, ERCC6L, CDT1, CHEK1, SPAG5, CENPH, SPC24, NUP37, BLM, CENPE, BUB3, CDK2, FANCD2, CENPO, CENPF, BRCA1, DSN1, MKI67, NCAPG2, H2AFX, HMGB2, SUV39H1, CBX3, TUBG1, KNTC1, PPP1CC, SMC2, BANF1, NCAPD2, SKA2, NUP107, BRCA2, NUP85, ITGB3BP, SYCE2, TOPBP1, DMC1, SMC4, INCENP.
chromosomal region	94	0	RAD51, OIP5, CDK1, SPC25, CCNB1, BIRC5, NCAPG, ZWINT, MAD2L1, SKA3, NUF2, BUB1B, CENPA, SKA1, AURKB, NEK2, ESCO2, CENPW, HJURP, TTK, NDC80, CDCA5, BUB1, ZWILCH, CENPK, KIF2C, AURKA, DSCC1, CENPN, CDCA8, CENPM, PLK1, MCM6, ERCC6L, CDT1, HELLS, CHEK1, SPAG5, CENPH, PCNA, SPC24, CENPI, NUP37, FEN1, CENPL, BLM, KIF18A, CENPE, MCM4, BUB3, SUV39H2, MCM2, CDK2, PIF1, DNA2, CENPO, CENPF, CHEK2, DSN1, H2AFX, MCM7, SUV39H1, MTBP, CBX3, RECQL4, KNTC1, PPP1CC, CENPP, CENPQ, PTGES3, NCAPD2, DYNLL1, SKA2, HAT1, NUP107, MCM5, MCM3, MSH2, BRCA2, NUP85, SSB, ITGB3BP, DMC1, INCENP, THOC3, XPO1, APEX1, XRCC5, KIF22, DCLRE1A, SEH1L, XRCC3, NSMCE2, RAD21.
mitochondrial protein complex	152	0	MRPL47, DNA2, MRPL11, MRPL42, TOMM5, MRPL3, MRPL21, NDUFA9, PPIF, MRPL13, NDUFA12, MRPL15, MRPS35, MRPL12, COX5A, CHCHD3, PNPT1, MRPS16, MRPL37, MRPS30, MRPL51, UQCRH, MRPS11, UQCRHL, MRPL52, MRPS15, MRPS22, TOMM40, MRPS12, MRPS17, TIMM8B, MRPS10, NDUFB5, TIMM9, MRPL35, COX7A2, NDUFB3, TIMM10, TIMM50, MRPL17, COX6A1, TOMM22, MRPL9, APOO, NDUFB4, MRPL48, MRPL27, MRPL30, MRPS7, CYC1, HSD17B10, MRPL44, MRPS33, VDAC1, MRPL36, MRPL22, PDK1, MRPS24, MRPL2, COX5B, UQCRFS1, CHCHD1, COX7A2L, C15orf48, ROMO1, NDUFS6, NDUFAB1, MRPL19, IMMT, MRPS18C, MRPL39, NDUFB9, MTX1, MRPL46, MRPL50, SUPV3L1, NDUFB6, MRPL32, SDHB, NDUFA8, TIMM17A, DAP3, MRPL16, NDUFB1, NDUFB8, UQCRC1, NDUFC2, GRPEL2, MFN1, MRPS5, MRPL18, NDUFA1, NDUFS3, GRPEL1, NDUFS1, MRPL10, NDUFS5, NDUFA6, NDUFV2, TIMM17B, MRPS28, DNAJC19, MTX2, UQCRQ, NDUFB11, MRPL33, MRPS9, IMMP1L, C12orf65, MRPL40, NDUFC1, SUCLG1, NDUFB2, MRPS14, NDUFS8, PARK7, DLAT, MRPS18A, KIAA0391, MRPL53, TOMM6, UQCRB, COX4I1, NDUFA11, NDUFA7, TIMM13, MRPL34, NDUFA4, NDUFA3, MRPS2, MRPS21, BCS1L, MTG1, MRPL24, CLPX, MRPL38, CHCHD10, MRPL28, TIMM22, FOXRED1, TOMM40L, SDHD, PMPCB, MRPL43, MRPS26, MRPL20, MRPL41, MPV17L2, NDUFS4, NDUFA5, MRPS34, NDUFA13.
ribosome	143	0	MRPL47, MRPL11, MRPL42, DENR, MRPL3, MRPL21, ZC3H15, MRPL13, MRPL15, MRPS35, MRPL12, HSPA14, PNPT1, MRPS16, MRPL37, MRPS30, MRPL51, MRPS11, MRPL52, MRPS15, MRPS22, MRPS12, MRPS17, MRPS10, MRPL35, MRPL17, NAA10, MRPL9, MRPL48, MRPL27, MRPL30, MRPS7, RPLP0, RPL39L, MRPL44, MRPS33, MRPL1, RPS7, MRPL36, RSL24D1, APEX1, MRPS23, MRPL22, PTCD3, MRPS24, MRPL2, EIF2AK2, CHCHD1, RPL22L1, NDUFAB1, MRPL19, MRPS18C, MRPL39, RPL27, MRPL46, MRPL50, MRPL32, DAP3, RPL26L1, MRPL16, RPL35A, RPL38, MRPS5, MRPL18, RPS19, RPS27A, LARP4, MRPL10, GADD45GIP1, RPL39, RPS16, MRPS28, RPS17, RPS10, RPL35, MCTS1, RPS21, NUFIP1, MRPL33, MRPS9, RPS3, C12orf65, MRPL40, RPS26, MRPS14, NCK1, RPSA, EIF2A, MRPS18A, MRPL53, RPL36A, RPS29, RPS18, RPL4, RPL7L1, RPL37, RPL24, RPL6, RPL8, RPL41, NDUFA7, MRPL34, RPL19, RPL23A, AURKAIP1, MRPS2, MRPS21, MTG1, MRPL24, ZNF622, MRPL38, RPS5, RPL30, MRPL28, RPLP1, RPS24, RPS15A, RPL31, EIF3H, MRPL43, NR0B1, RPS8, MRPS26, MRPL20, RPL18A, MRPL41, MPV17L2, MRPS34, RPL37A, RPL27A, RPL36AL, MRPL14, RPL18, RPL7A, RPS12, MRPL49, RPL5, RPS11, RPS15, RPS2, RPL23, RPL32, NSUN3.
spindle	70	0	CDK1, NUSAP1, CCNB1, BIRC5, MAD2L1, SKA3, KIF23, BUB1B, CDC6, PRC1, SKA1, AURKB, NEK2, TTK, CDC20, DLGAP5, KIF11, KIF20A, AURKA, RACGAP1, TPX2, KIFC1, KIF4A, CDCA8, PLK1, POC1A, CKAP2L, KIF15, SPAG5, SHCBP1, KIF18B, KIF14, ASPM, KIF18A, VRK1, ESPL1, ECT2, CENPE, TACC3, CKAP2, FAM83D, PSRC1, FBXO5, CDC7, KIF20B, CENPF, DSN1, CBX3, TUBG1, KNTC1, BCCIP, HAUS1, DYNLL1, SKA2, WDR62, RAE1, NUP85, TOPBP1, INCENP, HAUS2, HAUS6, MAPRE1, MAD2L2, HAUS8, KIF22, POC1B, CDC27, PRPF19, NEDD1, RAB11A.

Abbreviations: LeadingEdgeNum, the number of leading edge genes; FDR, false discovery rate from Benjamini and Hochberg from gene set enrichment analysis (GSEA).

Supplementary Table 3. Significantly enriched gene ontology ()GO annotations (biological processes) of KIAA0101 in lung adenocarcinoma (LinkedOmics).

Description	Leading EdgeNum	FDR	Leading Edge Gene
chromosome segregation	97	0	OIP5, NUSAP1, SPC25, CCNB1, BIRC5, NCAPG, ZWINT, MAD2L1, SKA3, NUF2, KIF23, BUB1B, CDC6, PRC1, SKA1, AURKB, NEK2, ESCO2, CENPW, CEP55, HJURP, TTK, CDC20, DLGAP5, NDC80, CDCA5, NCAPH, BUB1, KIF2C, RACGAP1, DSCC1, KIFC1, KIF4A, CENPN, CDCA8, TOP2A, PLK1, CDT1, SPAG5, RAN, KIF18B, KIF14, PTTG1, NUP37, FEN1, TRIP13, BLM, KIF18A, ESPL1, ECT2, CCNE2, EME1, CENPE, TACC3, FAM83D, BUB3, PSRC1, FBXO5, FANCD2, CENPF, BRCA1, DSN1, CCNE1, MKI67, BRIP1, TUBG1, KIF4B, CENPQ, ACTR3, SMC2, GEN1, NCAPD2, SKA2, NAA50, SYCE2, NAA10, DMC1, SMC4, INCENP, RAD51C, RAD18, RMI1, SRPK1, ANAPC5, MAD2L2, KIF22, KPNB1, FANCM, PHB2, SEH1L, XRCC3, CDC27, NSMCE2, RAD21, ANAPC11, RCC1, RAB11A.
DNA replication	96	0	RAD51, CDK1, CDC45, RRM2, CCNA2, CDC6, EXO1, ESCO2, MCM10, GINS1, GINS2, DSCC1, POLE2, MCM6, DTL, CDT1, CHEK1, RFC4, TIPIN, PCNA, DBF4, FEN1, BLM, GMNN, RNASEH2A, RFC5, RFC3, CCNE2, EME1, POLQ, MCM4, GINS4, RFC2, GINS3, MCM2, PRIM1, CDK2, FBXO5, CDC7, PIF1, DNA2, BRCA1, CHEK2, E2F8, CCNE1, POLA2, CHAF1B, BRIP1, WDHD1, DONSON, MCM7, TIMELESS, CLSPN, E2F7, RECQL4, SSBP1, SLBP, HMGA1, DUT, RPA3, CHAF1A, GEN1, STOML2, ATAD5, RRM1, RNASEH1, MCM5, MCM3, BRCA2, MCM8, GTPBP4, POLE3, DBF4B, RBBP7, RMI1, MSH6, SET, FANCM, RFWD3, POLD2, DNAJC2, PRIM2, NBN, FAF1, PPP2CA, KIN, CDK2AP1, RBBP8, POLE, SSRP1, LIG1, ATF1, POLD3, ZRANB3, DDX11, CDC34.
cell cycle checkpoint	60	0	CDK1, CCNB1, ZWINT, MAD2L1, CDC45, BUB1B, CDC6, AURKB, CDC25C, TTK, CDC20, NDC80, BUB1, ZWILCH, AURKA, GTSE1, TOP2A, PLK1, DTL, CDT1, CHEK1, TIPIN, PCNA, WDR76, TRIP13, BLM, EME1, BUB3, CDK2, E2F1, DNA2, CENPF, BRCA1, CHEK2, E2F8, BRIP1, H2AFX, DONSON, TIMELESS, CLSPN, E2F7, TRIAP1, KNTC1, GEN1, MSH2, PRMT1, TOPBP1, MSH6, MAD2L2, XRCC3, RFWD3, PRPF19, RINT1, TIPRL, NAE1, INTS7, NBN, ZNF207, PSMG2, TFDP1.
double-strand break repair	61	0	RAD51, CDC45, EXO1, ESCO2, RAD51AP1, CDCA5, GINS2, RAD54L, FOXM1, CHEK1, FEN1, TRIP13, BLM, EME1, RAD54B, PSMD14, FANCB, POLQ, GINS4, XRCC2, CDC7, DNA2, BRCA1, CHEK2, BRIP1, H2AFX, TIMELESS, UBE2N, RECQL4, UBE2V2, RPA3, GEN1, MSH2, BRCA2, MCM8, DMC1, SUMO1, RAD51C, PARP2, YY1, FIGNL1, RMI1, XRCC5, MAD2L2, PPP4C, DCLRE1A, DEK, XRCC3, RFWD3, NSMCE2, RAD21, PRPF19, DDX1, NBN, APTX, SFPQ, RBBP8, RECQL, TDP1, KDM1A, TDP2.
spindle organization	44	0	SPC25, CCNB1, NUF2, KIF23, PRC1, AURKB, NEK2, TTK, CDC20, NDC80, KIF11, AURKA, RACGAP1, TPX2, MYBL2, KIFC1, KIF4A, PLK1, POC1A, SPAG5, RAN, ASPM, ESPL1, CENPE, TACC3, STIL, PSRC1, FBXO5, CHEK2, STMN1, TUBB, TUBG1, KIF4B, BCCIP, HAUS1, WDR62, RAE1, HAUS2, HAUS6, MAPRE1, HAUS8, KPNB1, RCC1, RAB11A.

Abbreviations: LeadingEdgeNum, the number of leading edge genes; FDR, false discovery rate from Benjamini and Hochberg from gene set enrichment analysis (GSEA).

Supplementary Table 4. Significantly enriched gene ontology (GO) annotations (molecular functions) of KIAA0101 in lung adenocarcinoma (LinkedOmics).

Description	Leading Edge Num	FDR	Leading Edge Gene
structural constituent of ribosome	109	0	MRPL47, MRPL11, MRPL42, MRPL3, MRPL21, MRPL13, MRPL15, MRPS35, MRPL12, MRPS16, MRPL37, MRPS30, MRPL51, MRPS11, MRPL52, MRPS15, MRPS22, MRPS12, MRPS17, MRPL35, MRPL17, MRPL9, MRPL27, MRPL30, MRPS7, RPLP0, RPL39L, MRPS33, MRPL1, RPS7, MRPL36, RSL24D1, MRPS23, MRPL22, MRPS24, MRPL2, RPL22L1, MRPL19, MRPS18C, RPL27, MRPL46, MRPL32, DAP3, RPL26L1, MRPL16, RPL35A, RPL38, MRPS5, MRPL18, RPS19, RPS27A, MRPL10, RPL39, RPS16, RPS17, RPS10, RPL35, RPS21, MRPL33, MRPS9, RPS3, RPS26, MRPS14, RPSA, MRPS18A, RPL36A, RPS29, RPS18, RPL4, RPL7L1, RPL37, RPL24, RPL6, RPL8, RPL41, NDUFA7, MRPL34, RPL19, RPL23A, MRPS2, MRPS21, MRPL24, RPS5, RPL30, MRPL28, RPLP1, RPS24, RPS15A, RPL31, MRPL43, RPS8, MRPL20, RPL18A, MRPL41, MRPS34, RPL37A, RPL27A, RPL36AL, MRPL14, RPL18, RPL7A, RPS12, MRPL49, RPL5, RPS11, RPS15, RPS2, RPL23, RPL32.
catalytic activity, acting on DNA	69	0	RAD51, CDC45, EXO1, GINS1, GINS2, TOP2A, POLE2, MCM6, RAD54L, ERCC6L, PCNA, NEIL3, FEN1, BLM, NME1, EME1, RAD54B, POLQ, MCM4, GINS4, UNG, PIF1, DNA2, POLA2, BRIP1, MCM7, TDG, RECQL4, DKC1, HMGA1, PTGES3, GEN1, ALKBH2, RUVBL1, DMC1, POLE3, DNMT3B, RAD51C, APEX1, XRCC5, DCLRE1A, SMUG1, FANCM, XRCC3, POLE4, RUVBL2, POLD2, SUPV3L1, DDX1, DHX36, NBN, APTX, TATDN1, RBBP8, RECQL, POLE, TERT, LIG1, TDP1, CHRAC1, POLD3, TDP2, ZRANB3, METTL4, DDX11, G3BP1, RPS3, POLB, APEX2.
single-stranded DNA binding	43	0	RAD51, CDC45, MCM10, RAD51AP1, MCM6, NEIL3, BLM, NME1, MCM4, PRIM1, HMGB2, HSPD1, MCM7, RECQL4, SSBP1, RPA3, SMC2, GEN1, POLR2H, MSH2, TSN, BRCA2, YBX1, POLR2D, DMC1, SMC4, SUB1, POLR2G, RAD18, NUP35, LRPPRC, DHX36, PRIM2, HMGB1, APTX, CNBP, RAD23B, RBBP8, WBP11, TDP1, TDP2, POT1, DDX11.
helicase activity	43	0	CDC45, GINS1, GINS2, MCM6, RAD54L, ERCC6L, HELLS, BLM, RAD54B, MCM4, GINS4, MCM2, PIF1, DNA2, BRIP1, MCM7, EIF4A3, RECQL4, MCM5, MCM3, RUVBL1, MCM8, DDX47, DDX52, TTF2, DDX55, EIF4A1, XRCC5, FANCM, RUVBL2, DDX18, SUPV3L1, DDX1, DHX36, NBN, DDX56, HLTF, RECQL, GTF2F2, DDX23, DDX10, ZRANB3, DDX11.
catalytic activity, acting on RNA	122	0	EXO1, FEN1, RNASEH2A, RAD54B, PRIM1, PIF1, FARSB, CPSF3, EIF4A3, RPP30, EXOSC2, POP7, PNPT1, TARS, PTRH2, METTL2A, EXOSC8, RNASEH1, DARS, POLR2H, MARS, POP1, TSN, YARS2, EXOSC3, EMG1, DUS4L, POLR2D, POLR3G, FBL, EXOSC9, POLR2K, MRPL44, POLR2G, RPP40, GARS, DARS2, EIF4A1, APEX1, PUS1, EXOSC1, NOP2, METTL8, RPP25, DDX18, TRMT61B, SUPV3L1, POLR2J, ZNRD1, GATC, TRMT112, POP5, RARS, DDX1, METTL1, POLR1C, DHX36, PRIM2, METTL2B, POLR2I, DDX56, PPP1R8, TSEN15, POLR2F, IARS, METTL6, RBMX2, RPP38, TERT, CARS, POP4, DDX23, EXOSC5, DDX10, TDP2, NSUN2, KARS, FTSJ1, MARS2, EXOSC4, RPP21, G3BP1, EDC3, POLR3F, DTD1, NARS, ERI1, KIAA0391, MED20, WDR4, DHX37, DHX15, RNASEH2B, QRSL1, POLR3K, TWISTNB, ERI3, TRIT1, NARS2, TRMT12, TRPT1, DDX21, FARSA, YARS, WARS, SARS2, TFB2M, CNOT7, TRMT5, TGS1, DCPS, PUS3, ISG20L2, POLR2B, THUMPD3, DBR1, CDKAL1, THUMPD2, TFB1M, RARS2, EXOSC10, POLR3D.

Abbreviations: LeadingEdgeNum, the number of leading edge genes; FDR, false discovery rate from Benjamini and Hochberg from gene set enrichment analysis (GSEA).

Supplementary Table 5. Significantly enriched Kyoto Encyclopedia of Genes and Genomes (KEGG) pathway annotations of KIAA0101 in lung adenocarcinoma (LinkedOmics).

Description	Leading EdgeNum	FDR	Leading Edge Gene
Cell cycle	48	0	CCNB2, CDK1, CCNB1, MAD2L1, CDC45, BUB1B, CCNA2, CDC6, CDC25C, TTK, CDC20, BUB1, CDC25A, PLK1, MCM6, CHEK1, PCNA, DBF4, PTTG1, ESPL1, PKMYT1, CCNE2, MCM4, BUB3, MCM2, CDK2, E2F1, CDC7, CHEK2, CCNE1, E2F2, MCM7, ANAPC7, YWHAQ, SKP2, MCM5, MCM3, HDAC2, CDK4, YWHAZ, ANAPC5, MAD2L2, YWHAG, RBL1, CDC27, E2F3, RAD21, ANAPC11.
Ribosome	100	0	MRPL11, MRPL3, MRPL21, MRPL13, MRPL15, MRPL12, MRPS16, MRPS11, MRPS15, MRPS12, MRPS17, MRPS10, MRPL35, MRPL17, MRPL9, MRPL27, MRPL30, MRPS7, RPLP0, MRPL1, RPS7, MRPL36, RSL24D1, MRPL22, MRPL2, RPL22L1, MRPL19, MRPS18C, RPL27, MRPL32, RPL26L1, MRPL16, RPL35A, RPL38, MRPS5, MRPL18, RPS19, RPS27A, MRPL10, RPL39, RPS16, RPS17, RPS10, RPL35, RPS21, MRPL33, MRPS9, RPS3, RPS26, MRPS14, RPSA, MRPS18A, RPL36A, RPS29, RPS18, RPL4, RPL37, RPL24, RPL6, RPL8, RPL41, MRPL34, RPL19, RPL23A, MRPS2, MRPS21, MRPL24, FAU, RPS5, RPL30, MRPL28, RPLP1, RPS24, RPS15A, RPL31, RPS8, MRPL20, RPL18A, RPL37A, RPL27A, RPL36AL, MRPL14, RPL18, RPL7A, RPS12, RPL5, RPS11, RPS15, RPS2, RPL23, RPL32, UBA52, RPL36, MRPL4, RPL17, RPS20, RPS3A, RPL29, RPS13, RPL10L.
Proteasome	40	0	PSMD14, PSMA4, PSMD12, PSMA5, PSMB3, PSMA2, PSMB7, POMP, PSMB5, PSMA3, PSMD11, PSMC4, PSMA7, PSMC6, PSMA1, PSMA6, PSMC2, PSMB4, PSME2, PSMC1, PSMB2, PSMB1, PSME3, PSMD3, PSMB6, PSMC3, PSMD7, PSMD2, PSMD13, PSMD4, PSMD8, PSMD1, PSMD6, PSME4, ADRM1, IFNG, PSMB8, PSMB9, PSMC5, PSME1.
Spliceosome	63	0	SNRPA1, SNRPD1, SNRPF, SNRPG, LSM5, SNRPB, HNRNPC, MAGOHB, EIF4A3, LSM2, SMNDC1, SNRPE, PPIL1, SNRPC, PHF5A, PPIH, MAGOH, SNRPD2, PRPF4, SNRPB2, LSM4, LSM3, SNRNP27, SNRPA, TRA2B, BUD31, THOC3, RBM17, USP39, PRPF40A, EFTUD2, SNRPD3, PRPF19, LSM6, ISY1, SNRNP40, CWC15, NCBP1, WBP11, DDX23, LSM7, NCBP2, TXNL4A, SF3B5, BCAS2, U2AF2, PUF60, RBMX, HNRNPK, SNW1, U2AF1, PLRG1, DHX15, PCBP1, SF3B4, HNRNPA1, SF3A3, CDC5L, PRPF38A, TCERG1, PQBP1, HSPA1B, HNRNPA1L2.
DNA replication	27	0	POLE2, MCM6, RFC4, PCNA, FEN1, RNASEH2A, RFC5, RFC3, MCM4, RFC2, MCM2, PRIM1, DNA2, POLA2, MCM7, SSBP1, RPA3, RNASEH1, MCM5, MCM3, POLE3, POLE4, POLD2, PRIM2, POLE, LIG1, POLD3.

Abbreviations: LeadingEdgeNum, the number of leading edge genes; FDR, false discovery rate from Benjamini and Hochberg from gene set enrichment analysis (GSEA).

Supplementary Table 6. Significantly enriched kinase-target networks of KIAA0101 in lung adenocarcinoma (LinkedOmics).

Description	Leading EdgeNum	FDR	Leading Edge Gene
Kinase_CDK1	74	0	NUSAP1, CCNB1, BIRC5, PBK, NCAPG, RRM2, BUB1B, CENPA, PRC1, CDC25C, CEP55, CDC20, DLGAP5, CDCA5, BUB1, KIF11, KIF2C, TPX2, TK1, TOP2A, CDC25A, DTL, FOXM1, ERCC6L, CHEK1, RFC4, SPAG5, FEN1, BLM, ESPL1, UHRF1, RFC5, RFC3, ECT2, NME1, CKAP2, TMPO, RFC2, E2F1, UNG, CDC7, KIF20B, EZH2, LMNB1, BRCA1, PAICS, MKI67, STMN1, FANCG, EIF4EBP1, MCM7, GMPS, SLBP, MAPK6, HMGA1, DUT, NME2, BRCA2, LDHA, ZC3HC1, FBXO43, LMNB2, XPO1, KIF22, USP14, DNM1L, CDC27, ANAPC11, NEDD1, CSNK2B, RCC1, LBR, USP1, PPP1CA.
Kinase_PLK1	32	0	RAD51, CCNB1, BIRC5, BUB1B, CDC6, PRC1, CDC25C, CEP55, KIF2C, RACGAP1, GTSE1, TOP2A, CDC25A, FOXM1, ERCC6L, RAN, ESPL1, PKMYT1, STIL, FBXO5, BRCA1, CHEK2, CLSPN, ANAPC7, CENPQ, RUVBL1, BRCA2, FBXO43, YY1, CDC27, NEDD1, SUZ12.
Kinase_AURKB	35	0	NUSAP1, BIRC5, KIF23, CENPA, AURKB, NDC80, CDCA5, KIF2C, RACGAP1, CDCA2, KIF4A, CDCA8, PLK1, SHCBP1, CKAP2, DSN1, MKI67, HIST1H3B, DDX52, INCENP, PPHLN1, YY1, CCDC86, DEK, HIST1H3C, HIST1H3I, HMGN2, MPHOSPH10, HIST1H3F, NSUN2, RPS10, HIST1H3G, HIST1H3J, RBMX, KRT8.
Kinase_CDK2	73	0	RRM2, CCNA2, CDC6, CDC25C, CDC20, DLGAP5, NCAPH, TPX2, MYBL2, TK1, DTL, FOXM1, CDT1, CHEK1, DIAPH3, BLM, UHRF1, MCM4, MCM2, CDK2, E2F1, UNG, CDC7, EZH2, CENPF, BRCA1, CCNE1, PAICS, MKI67, E2F2, STMN1, MCM7, GMPS, EIF4A3, TUBG1, C9orf40, ANAPC7, HMGA1, NUP107, SKP2, C2orf49, MCM3, TSN, BRCA2, TFAM, ITGB3BP, ZC3HC1, TOPBP1, LMNB2, RAD18, ANAPC5, KIF22, RBL1, CEP76, DNM1L, CDC27, E2F3, ANAPC11, CSNK2B, ERAL1, MTHFD1L, TBCE, PPP1CA, NBN, PYCR1, NPM1, SCML2, RBBP8, HIST1H1E, ANAPC10, MTA2, LIG1, TSR1.
Kinase_ATR	20	0	FANCI, GINS2, CHEK1, DBF4, BLM, MCM2, E2F1, FANCD2, BRCA1, CHEK2, H2AFX, CLSPN, FANCA, MCM3, DCK, XRCC3, NBN, NPM1, RBBP8, TDP1.

Abbreviations: LeadingEdgeNum, the number of leading edge genes; FDR, false discovery rate from Benjamini and Hochberg from gene set enrichment analysis (GSEA).

Supplementary Table 7. Significantly enriched miRNA-target networks of KIAA0101 in lung adenocarcinoma (LinkedOmics).

Description	Leading EdgeNum	FDR	Leading Edge Gene
GAGCCTG,MIR-484	40	0.010236	DPYSL2, EZH1, DLEC1, HSPG2, PTPRE, PLEKHH2, FOXO4, SCARA3, MYCBP2, HIPK1, PITPNA, NFIA, ZFYVE1, LBH, HTT, EDA, PRKCB, PTGER4, PRRT2, SLC6A1, TAF1L, SORBS2, FAM13A, FRMPD4, DACH1, TRIOBP, ZYG11B, KLF12, BCL11A, GAPVD1, KDM4A, MINK1, MAPKAPK2, HIVEP2, DENND5A, SNN, ACVR1B, HLA-DOB, PTPRF, WDR90.
CAGCACT,MIR-512-3P	53	0.025589	HLF, PLEKHM1, TAL1, RTN4RL1, ATXN7, C1orf21, SGSM2, TLN1, TRIM3, UBL3, ATXN1, PTPRT, FOXN3, FEM1C, MLLT6, IP6K1, RNF38, TNRC6B, ETV1, GRM7, 8-Mar, PPP3CA, USP47, TP53INP1, ESRP2, TRHDE, MBNL2, PDIK1L, PCDHAC2, TRIM2, PCDHA10, PPFIA2, NTNG1, GIGYF1, KLHL3, ARHGEF3, RSBN1, MRPS25, HCN4, SYT8, ARHGEF10, NFIB, ZDHHC9, NEO1, BHLHE41, PCDHA3, PCDH10, FRMD4A, BAHD1, KCNRG, SLC2A4, XIAP, CDK19.
TTTGCAC,MIR-19A,MIR-19B	148	0.029684	ZBTB4, CBX7, ADCY9, RAI2, DLC1, ROBO2, PRICKLE2, SMARCA2, TGFBR2, HLF, SCARF1, MACF1, ST3GAL5, CACNA1C, RALGPS1, ERBB4, CNTFR, PARM1, WDFY3, RFX1, ZDHHC7, LRIG1, ARHGEF12, NCALD, PTK2B, RGL1, KLF13, RXRA, ATP11A, CYLD, MECP2, SRGAP3, KCNA4, RBMS3, FOXF2, OLFM1, ARHGAP1, MYLIP, ABR, SPEN, UBL3, ATXN1, ARC, SPRYD3, SH3D19, STAT5B, AFF1, TMEM63B, ATRX, HIPK1, FEM1C, CPEB4, TNRC6A, SLC24A4, MLLT6, RNF145, CREBL2, FOXP1, RTN1, RNF38, NBEA, TNRC6B, KCNS2, KLHL20, ETV1, MEF2D, S1PR1, SOX6, PTPRG, DLX3, LBH, TSC1, GRIN2A, INO80, CCND2, TGOLN2, SYBU, SLC9A1, GRM7, HECW2, ZEB2, PDE7B, MED26, ITPR1, ATP10A, MAGI2, BACE1, ADCY7, BMPR2, TRAK2, WDR47, LRRK1, PRRT3, TP53INP1, ARID4B, AKAP1, ESRP2, ANKRD12, PHLDA3, PDE5A, KIF3A, ZFPM2, ZFYVE26, COL19A1, EVI5L, ENPP5, SHANK2, EPC2, PLXNC1, BPTF, ARRDC4, MBNL2, MID1IP1, SMOC2, KIAA1217, ZMYND11, ARRDC3, UCP3, PCDHAC2, PHF12, MFSD6, EPN2, WBP2, CGN, PPARA, PCDHA10, RIN2, FZD8, BSN, VGLL4, SDC1, ID4, SLC24A3, OGT, IGF2R, DDX6, IGSF3, SOX5, TESK2, PCDHA3, MINK1, ARGLU1, PCDH10, SLAIN1, BTBD7, NAV3, MAP3K12, PRUNE2.
GACAATC,MIR-219	60	0.034546	CGNL1, AKAP13, TGFBR2, ZCCHC24, CELF2, EPHA4, ERG, CBFA2T3, FAM160A2, PTPRU, SH3D19, DDAH1, CPEB3, NR2C2, TACC1, HAS3, KLF9, SNRK, ZC3H12B, MEF2D, SOX6, ETV5, MKNK2, PIP5K1C, GTPBP1, SYNGAP1, AFF4, MFNG, INPP5J, TRHDE, THRB, TMEM98, PODXL, AGPAT3, RECK, PDZRN4, FBXL17, NCOA1, NTNG1, PDGFRA, CXXC5, PCDH17, ZNF827, EGR3, FZD4, BTBD7, FBXO3, ZNF609, CCDC28A, PHACTR2, MAPT, ERGIC1, CPEB2, SDK1, FAM120C, HOMER2, KIF1B, UBR1, RORB, FMNL2.
CCCAGAG,MIR-326	58	0.039237	PLXNA2, CORO2B, PTCH1, SCARF1, KIAA0513, CELF2, ANKFY1, NCALD, H6PD, ZMIZ1, TLN1, BSDC1, SMAD6, ATXN1, SPRYD3, RPS6KA1, SEMA6D, RPS6KA3, UBXN10, VPS39, CIC, GNAO1, AHCYL2, MMP24, PALM, DIDO1, PTK7, PPP1R9B, SYNGAP1, CEBPA, TCF4, ATP8B2, FAIM2, EPN2, GGT7, RPGR, OGT, C9orf24, LRRC32, SSH2, NAV3, ST3GAL3, PAPPA, ZNF609, KCNIP2, CRIM1, BRPF3, NRP1, LRRTM1, BCL11B, AGPAT4, DLGAP2, KLHL14, EGLN2, RALGAPA1, NHS, SEC63, ATP2B2.

Abbreviations: LeadingEdgeNum, the number of leading edge genes; FDR, false discovery rate from Benjamini and Hochberg from gene set enrichment analysis (GSEA).

Supplementary Table 8. Significantly enriched transcription factor-target networks of KIAA0101 in lung adenocarcinoma (LinkedOmics).

Description	Leading EdgeNum	FDR	Leading Edge Gene
E2F_Q6	87	0	RAD51, CDK1, CDC45, RRM2, CDC6, H2AFZ, POLE2, CDC25A, ARHGAP11A, MCM6, DNAJC9, CDT1, PCNA, GMNN, SNRPD1, PKMYT1, RANBP1, TMPO, MCM4, GINS3, MCM2, FBXO5, E2F1, FANCD2, UNG, ZNF367, EZH2, ATAD2, E2F8, STMN1, POLA2, GAPDH, MCM7, SASS6, CLSPN, E2F7, PPP1CC, EED, PHF5A, GEN1, ATAD5, WDR62, MCM3, MSH2, MCM8, TOPBP1, SUMO1, TRA2B, CDCA7, DCK, HMGXB4, HIST1H2AH, KPNB1, RBL1, POLE4, E2F3, MXD3, TRMT6, DCTPP1, INTS7, HMGN2, PPP1R8, CTDSPL2, KCND2, SYNGR4, POLD3, UXT, NOLC1, ZCCHC8, HNRNPR, SLC38A1, MRPL40, ACBD6, MTF2, NASP, PRPS1, MAZ, CAND1, SMC6, YBX2, HIST1H4A, EHBP1, HNRNPD, AP4M1, HNRNPA1, PRKDC, PCSK1.
VE2F_Q4	86	0	RAD51, CDK1, CDC45, RRM2, CDC6, H2AFZ, POLE2, CDC25A, ARHGAP11A, MCM6, DNAJC9, CDT1, PCNA, GMNN, SNRPD1, PKMYT1, RANBP1, TMPO, MCM4, GINS3, MCM2, FBXO5, E2F1, FANCD2, UNG, ZNF367, EZH2, ATAD2, E2F8, STMN1, POLA2, GAPDH, MCM7, SASS6, CLSPN, E2F7, PPP1CC, EED, PHF5A, GEN1, ATAD5, WDR62, MCM3, MSH2, MCM8, TOPBP1, SUMO1, TRA2B, CDCA7, DCK, HMGXB4, HIST1H2AH, KPNB1, RBL1, POLE4, E2F3, MXD3, TRMT6, DCTPP1, INTS7, HMGN2, PPP1R8, CTDSPL2, KCND2, SYNGR4, POLD3, UXT, NOLC1, ZCCHC8, HNRNPR, SLC38A1, MRPL40, ACBD6, MTF2, NASP, PRPS1, MAZ, CAND1, SMC6, YBX2, EHBP1, HNRNPD, AP4M1, HNRNPA1, PRKDC, PCSK1.
E2F4DP1_01	94	0	CDK1, RRM2, CDC6, H2AFZ, POLE2, CDC25A, ARHGAP11A, MCM6, DNAJC9, PCNA, GMNN, SNRPD1, PKMYT1, RANBP1, TMPO, MCM4, GINS3, MCM2, FBXO5, E2F1, FANCD2, UNG, ZNF367, EZH2, ATAD2, E2F8, STMN1, FANCG, GAPDH, MCM7, SASS6, CLSPN, SUV39H1, E2F7, CBX3, MAPK6, HMGA1, EED, PHF5A, GEN1, ATAD5, WDR62, MCM3, MSH2, MCM8, TOPBP1, SUMO1, TRA2B, CDCA7, DCK, HMGXB4, RBBP7, EIF4A1, HIST1H2AH, RBL1, POLE4, E2F3, MXD3, TRMT6, PRPS2, DCTPP1, CTDSPL2, LIG1, SYNGR4, POLD3, NOLC1, H2AFV, ZCCHC8, HNRNPR, AP1S1, MRPL40, ACBD6, MTF2, NASP, PRPS1, MAZ, CAND1, SMC6, YBX2, HIST1H4A, EHBP1, HNRNPD, HNRNPA2B1, AP4M1, PRKDC, PCSK1, FANCC, NCL, USP37, POLD1, CDC5L, DNMT1, IER5L, NUP62.
E2F1_Q6	96	0	CDK1, RRM2, CDC6, H2AFZ, POLE2, CDC25A, ARHGAP11A, MCM6, DNAJC9, CDT1, PCNA, GMNN, SNRPD1, PKMYT1, RANBP1, TMPO, MCM4, GINS3, MCM2, FBXO5, E2F1, FANCD2, UNG, ZNF367, EZH2, ATAD2, E2F8, STMN1, FANCG, GAPDH, MCM7, SASS6, CLSPN, SUV39H1, E2F7, CBX3, HMGA1, EED, PHF5A, GEN1, ATAD5, WDR62, MCM3, MSH2, MCM8, GPN3, TOPBP1, SUMO1, TRA2B, CDCA7, DCK, HMGXB4, HIST1H2AH, KPNB1, RBL1, SLC25A3, POLE4, E2F3, MXD3, TRMT6, PRPS2, DCTPP1, CTDSPL2, SYNGR4, POLD3, NOLC1, H2AFV, SERBP1, ZCCHC8, HNRNPR, AP1S1, MRPL40, ACBD6, MTF2, NASP, ZBTB8OS, PRPS1, MAZ, CAND1, SMC6, YBX2, HIST1H4A, EHBP1, HNRNPD, HNRNPA2B1, AP4M1, PRKDC, PCSK1, FANCC, NCL, USP37, POLD1, CDC5L, DNMT1, IER5L, NUP62.
E2F_02	93	0	CDK1, RRM2, CDC6, H2AFZ, POLE2, CDC25A, ARHGAP11A, MCM6, DNAJC9, PCNA, GMNN, SNRPD1, PKMYT1, RANBP1, TMPO, MCM4, GINS3, MCM2, FBXO5, E2F1, FANCD2, UNG, ZNF367, EZH2, ATAD2, E2F8, STMN1, FANCG, GAPDH, MCM7, SASS6, CLSPN, SUV39H1, E2F7, CBX3, MAPK6, HMGA1, EED, PHF5A, GEN1, ATAD5, WDR62, MCM3, MSH2, MCM8, PTMA, TOPBP1, SUMO1, TRA2B, CDCA7, DCK, HMGXB4, EIF4A1, HIST1H2AH, RBL1, POLE4, E2F3, MXD3, TRMT6, PRPS2, DCTPP1, CTDSPL2, SYNGR4, POLD3, NOLC1, H2AFV, ZCCHC8, HNRNPR, AP1S1, MRPL40, ACBD6, MTF2, NASP, PRPS1, MAZ, CAND1, SMC6, YBX2, HIST1H4A, EHBP1, HNRNPD, HNRNPA2B1, AP4M1, PRKDC, PCSK1, FANCC, NCL, USP37, POLD1, CDC5L, DNMT1, IER5L, NUP62.

Abbreviations: LeadingEdgeNum, the number of leading edge genes; FDR, false discovery rate from Benjamini and Hochberg from gene set enrichment analysis (GSEA).
