# Supplementary material for: KIAA0101 as a new diagnostic and prognostic marker, and its correlation with gene regulatory networks and immune infiltrates in lung adenocarcinoma
Source: Aging (Albany NY). 2020 Nov 20;13(1):301–39. doi: 10.18632/aging.104144 (PMC7835026; doi:10.18632/aging.104144)
Supplement: Supplementary Tables [file aging-13-104144-s002.pdf]

SUPPLEMENTARY FIGURES

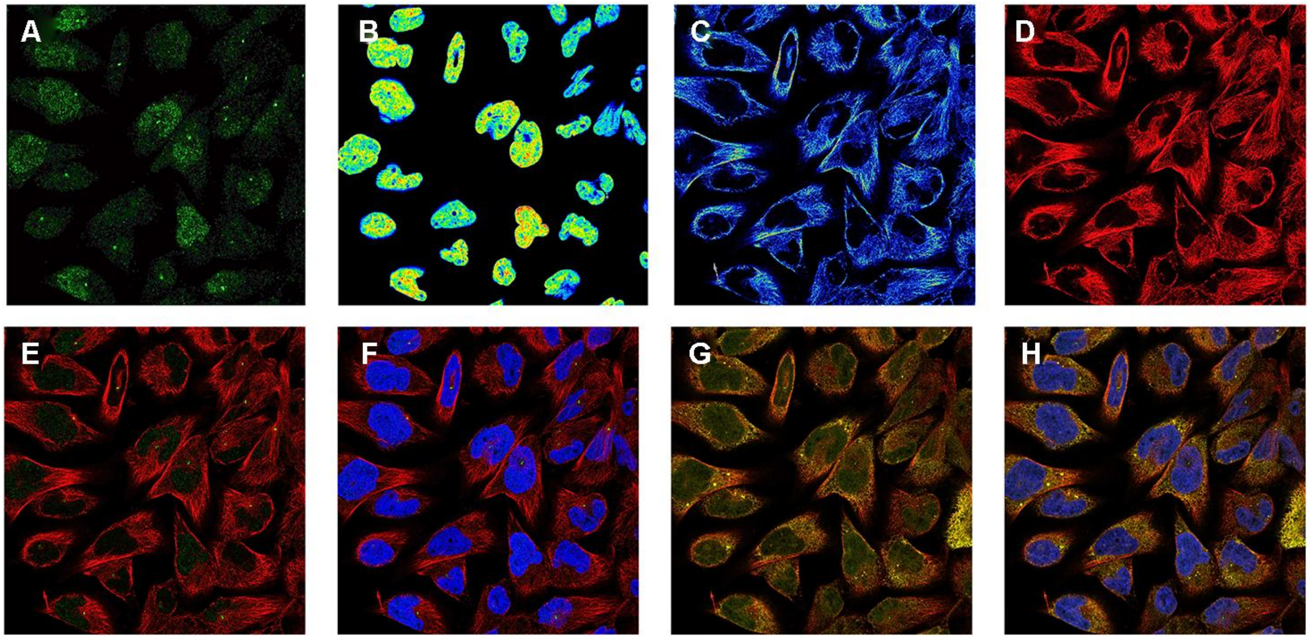

Supplementary Figure 1. Subcellular mapping of the KIAA0101 human proteome in lung adenocarcinoma cells. (**A**–**H**) Represent the subcellular mapping of the KIAA0101 human proteome (RH-30, HPA047929), respectively. (**A**) Antibody. (**B**) Nucleus and intensity. (**C**) Microtubule and intensity. (**D**) Microtubule. (**E**) Antibody and microtubule. (**F**) Antibody, nucleus, and microtubule. (**G**) Microtubule and endoplasmic reticulum. (**H**) Microtubule and endoplasmic reticulum. The cell structure sections were based on representative images of antibody-stained human cancer cell lines. Each sample was stained with an in-house generated HPA-antibody and counterstained with markers for microtubules, the endoplasmic reticulum, and the nucleus.

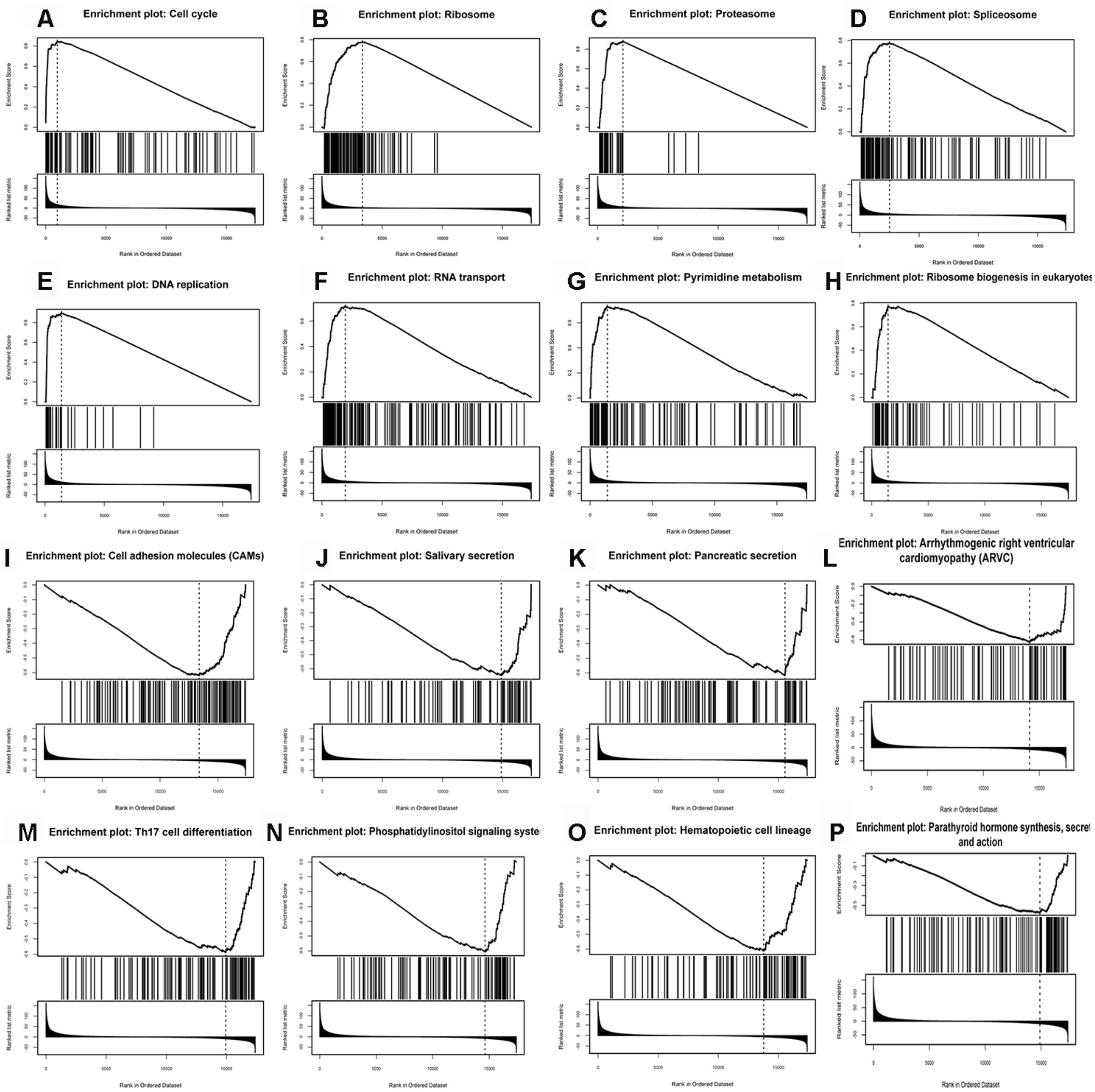

Supplementary Figure 2. Enrichment plot of pathways in the enrichment results of figure 3A with the top 8 and the bottom 8 genes according to the normalized enrichment score. (**A**) hsa04110, Cell cycle; (**B**) hsa03010, Ribosome; (**C**) hsa03050, Proteasome; (**D**) hsa03040, Spliceosome; (**E**) hsa03030, DNA replication; (**F**) hsa03013, RNA transport; (**G**) hsa00240, Pyrimidine metabolism; (**H**) hsa03008, Ribosome biogenesis in eukaryotes; (**I**) hsa04514, Cell adhesion molecules (CAMs); (**J**) hsa04970, Salivary secretion; (**K**) hsa04972, Pancreatic secretion; (**L**) hsa05412, Arrhythmogenic right ventricular cardiomyopathy (ARVC); (**M**) hsa04659, Th17 cell differentiation; (**N**) hsa04070, Phosphatidylinositol signaling system; (**O**) hsa04640, Hematopoietic cell lineage; (**P**) hsa04928, Parathyroid hormone synthesis secretion and action.

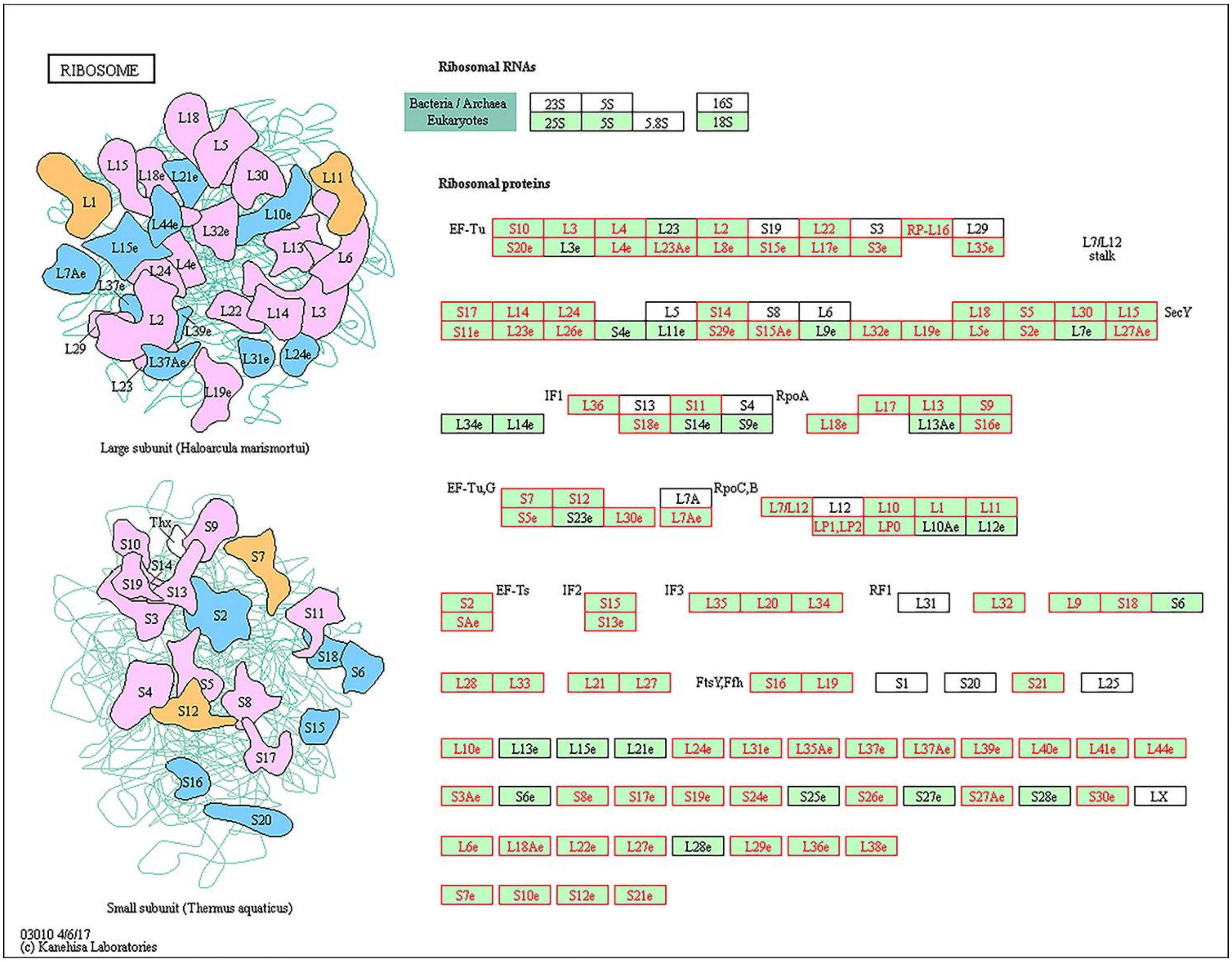

Supplementary Figure 3. KEGG pathway annotations of the ribosome pathway (HSA03010). Red denotes leading edge genes; green denotes the remaining genes.

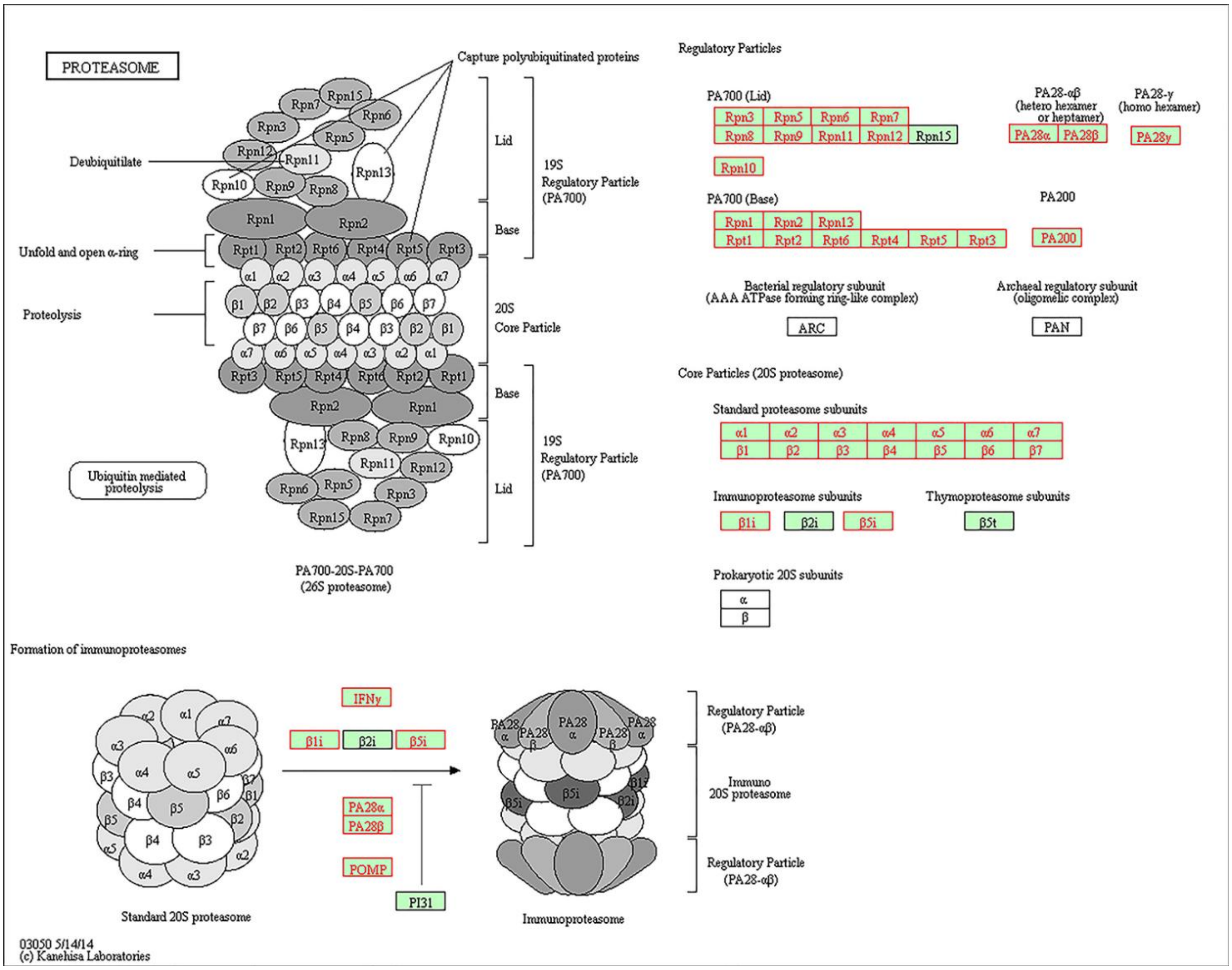

Supplementary Figure 4. KEGG pathway annotations of the Proteasome pathway (hsa03050). Red denotes leading edge genes; green denotes the remaining genes.

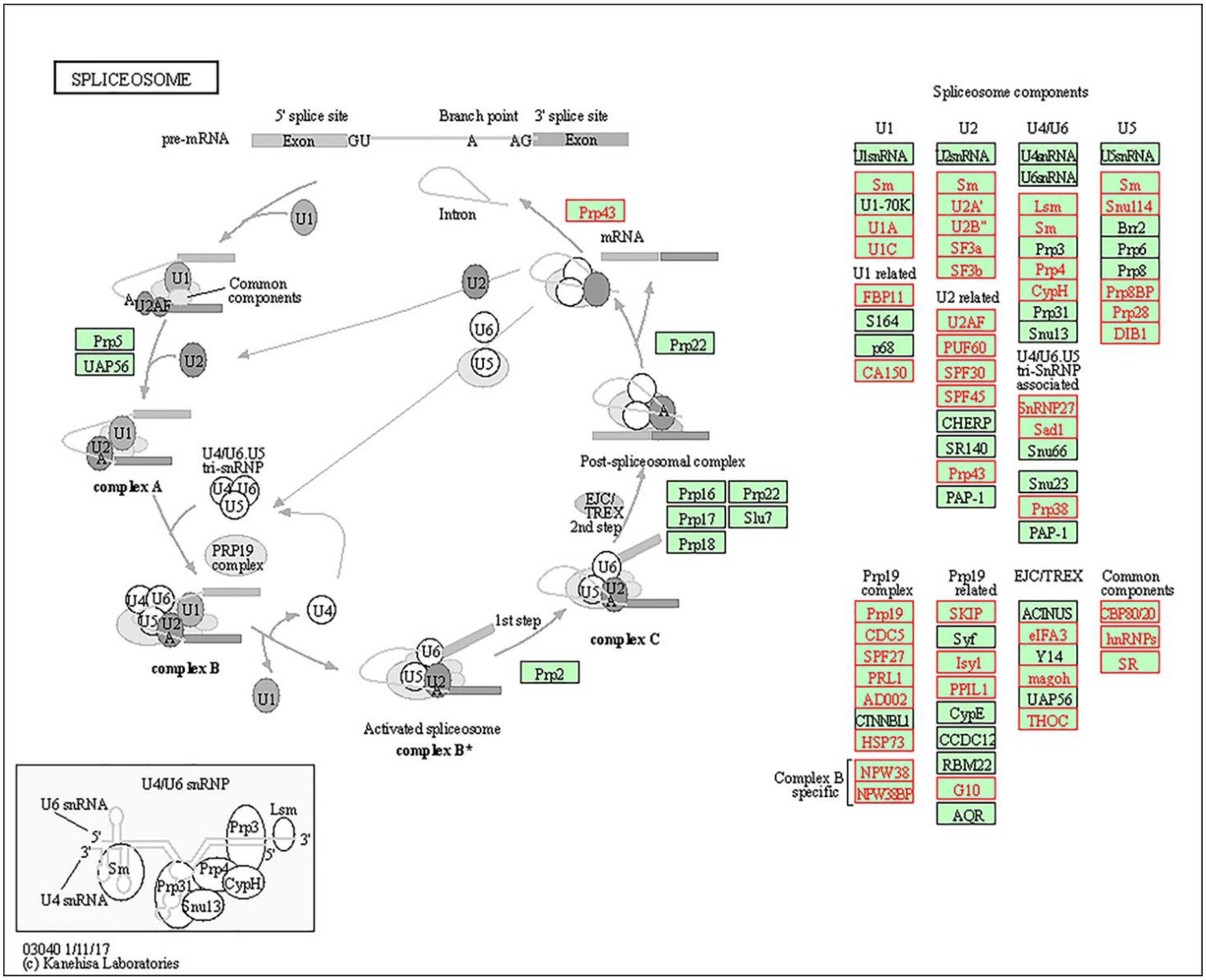

Supplementary Figure 5. KEGG pathway annotations of the Spliceosome pathway (hsa03040). Red denotes leading edge genes; green denotes the remaining genes.

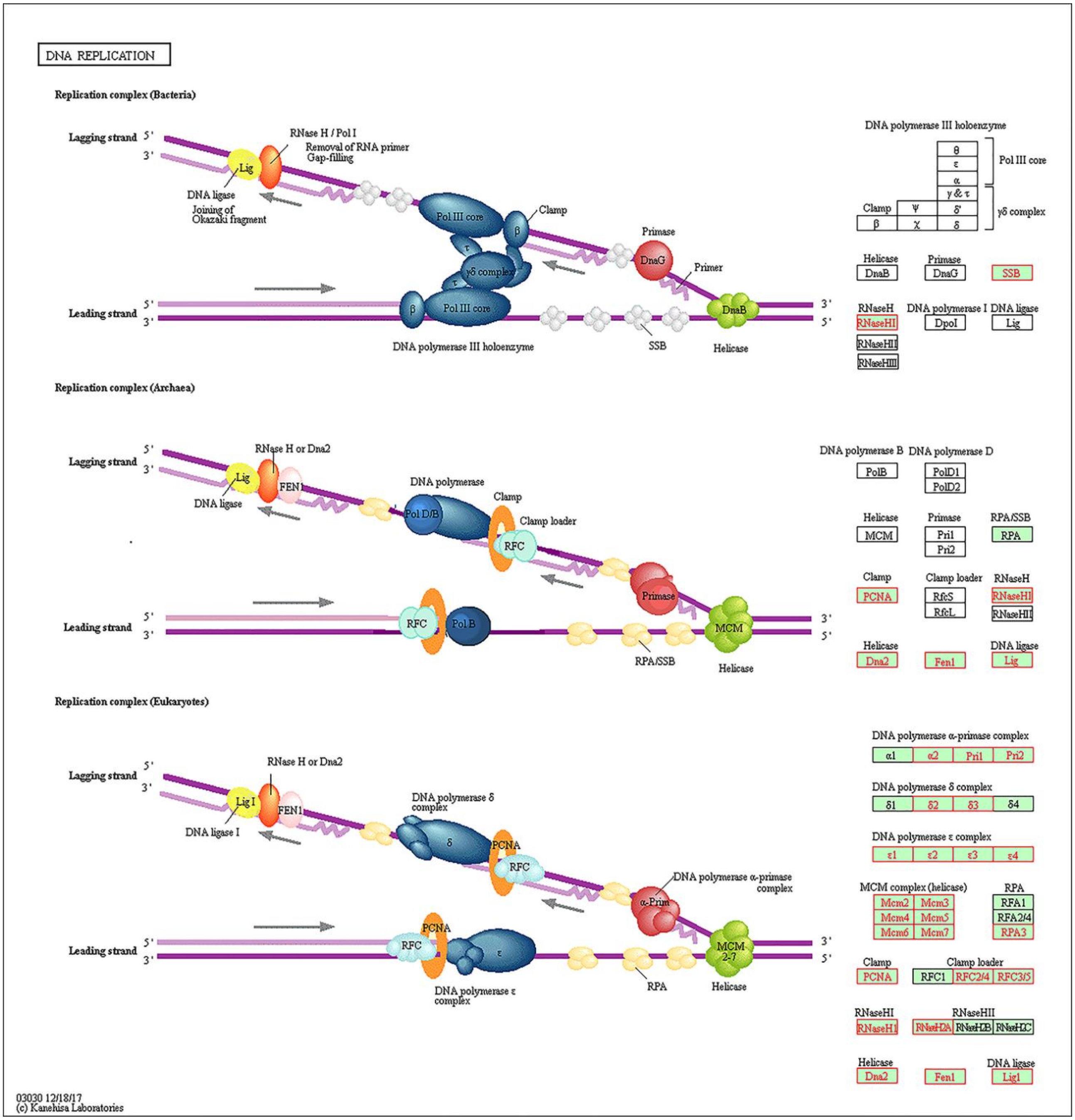

Supplementary Figure 6. KEGG pathway annotations of the DNA replication pathway (hsa03030). Red denotes leading edge genes; green denotes the remaining genes.

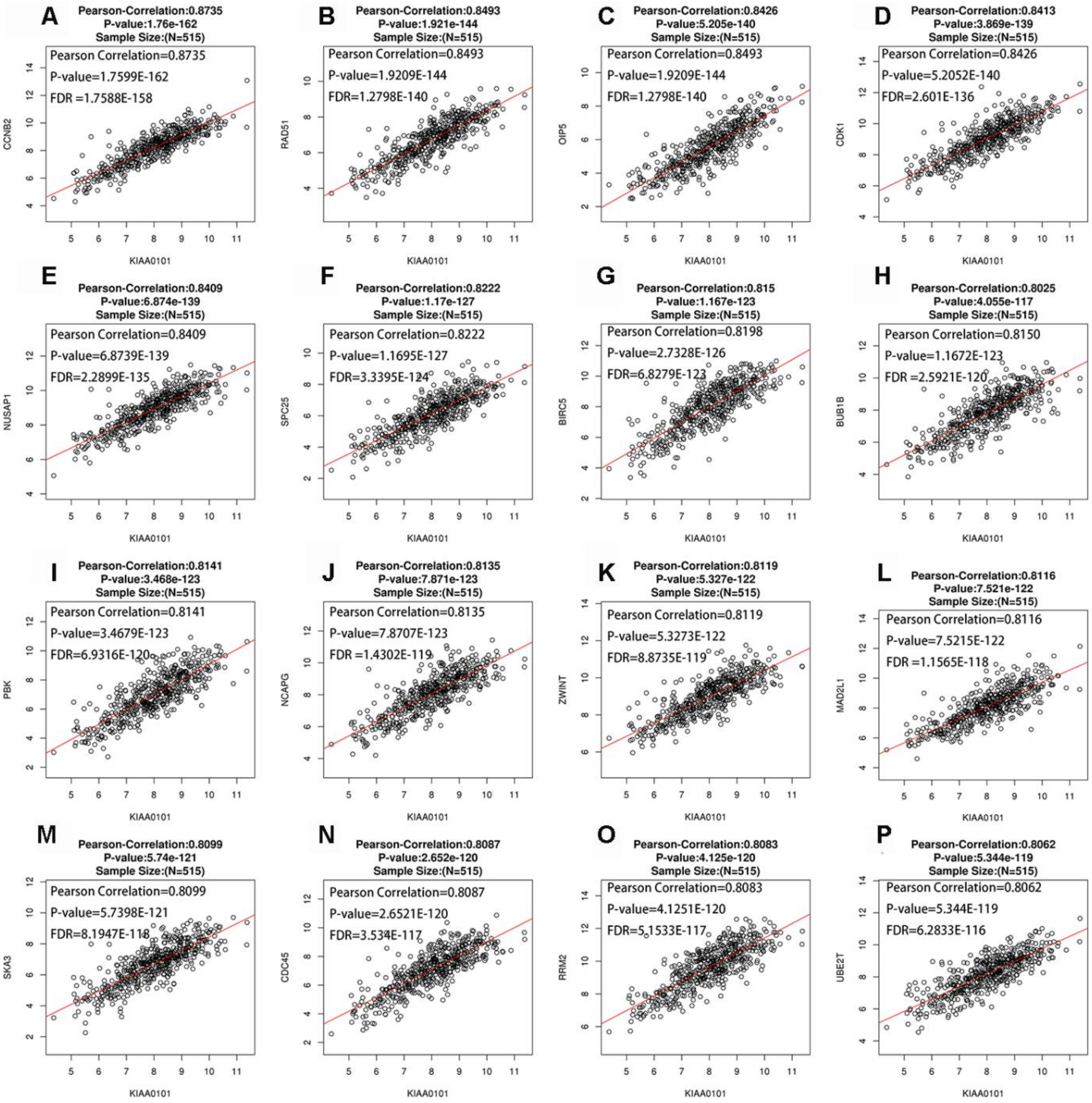

Supplementary Figure 7. The scatter diagram of *KIAA0101* and the first 16 genes highly related to *KIAA0101* (Pearson correlation coefficient > 0.8). (**A**) *CCNB2*; (**B**) *RAD51*; (**C**) *OIP5*; (**D**) *CDK1*; (**E**) *NUSAP1*; (**F**) *SPC25*; (**G**) *CCNB1*; (**H**) *BIRC5*; (**I**) *PBK*; (**J**) *NCAPG*; (**K**) *ZWINT*; (**L**) *MAD2L1*; (**M**) *SKA3*; (**N**) *CDC45*; (**O**) *RRM2*; (**P**) *UBE2T*.

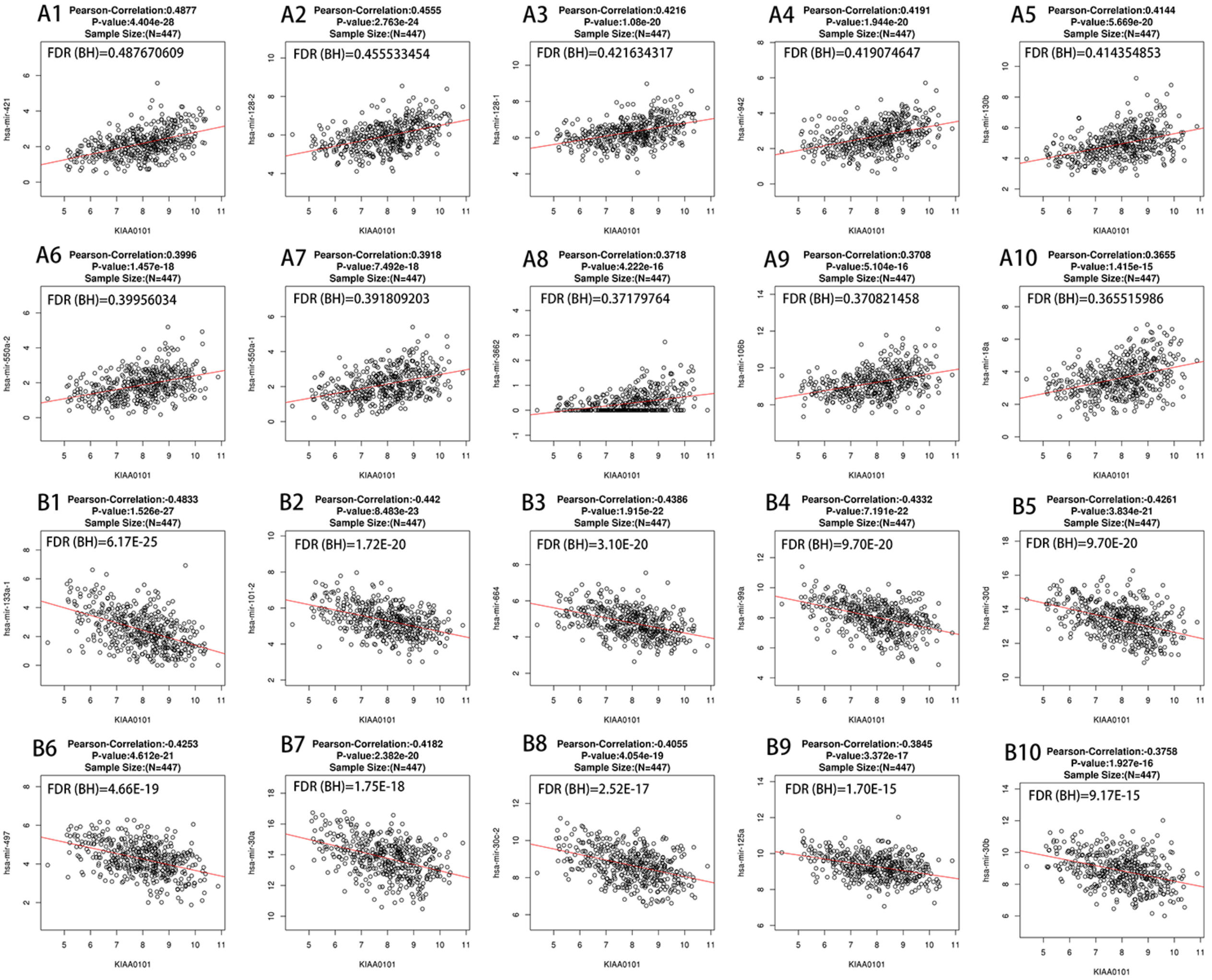

Supplementary Figure 8. Expression of *KIAA0101* and correlation scatter diagram of miRNAs. (**A1**–**A10**) Positive correlations between the expression of *KIAA0101* and miRNAs. (**B1**–**B10**) Negative correlations between the expression of *KIAA0101* and miRNAs.

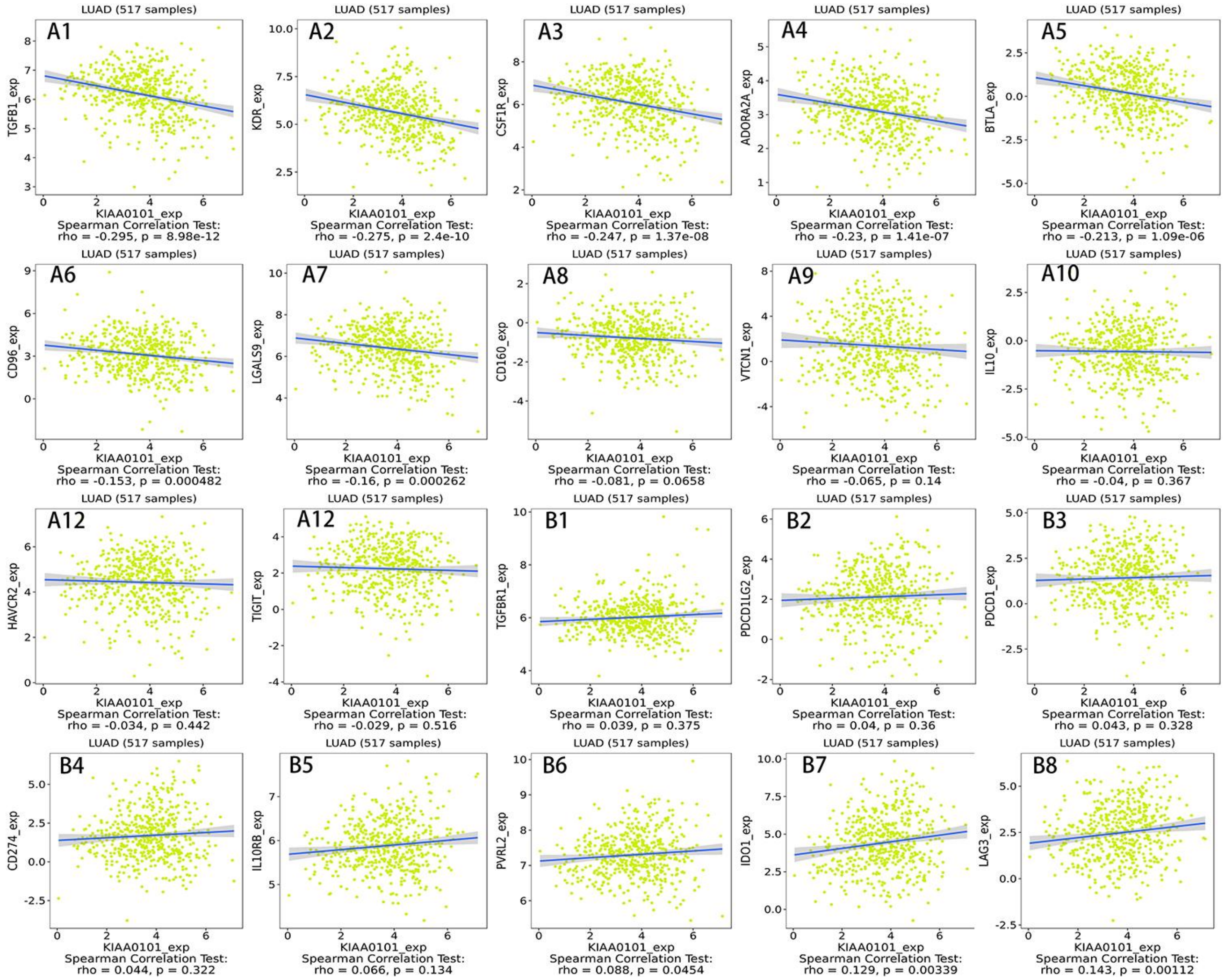

Supplementary Figure 9. Correlations between the expression of *KIAA0101* and immunoinhibitors. (**A1**–**A12**) are scatter plots of the negative correlations between *KIAA0101* expression and immunoinhibitors in the treatment of lung adenocarcinoma. (**B1**–**B8**) are scatter plots of the positive correlations between *KIAA0101* expression and immunoinhibitors in the treatment of lung adenocarcinoma.

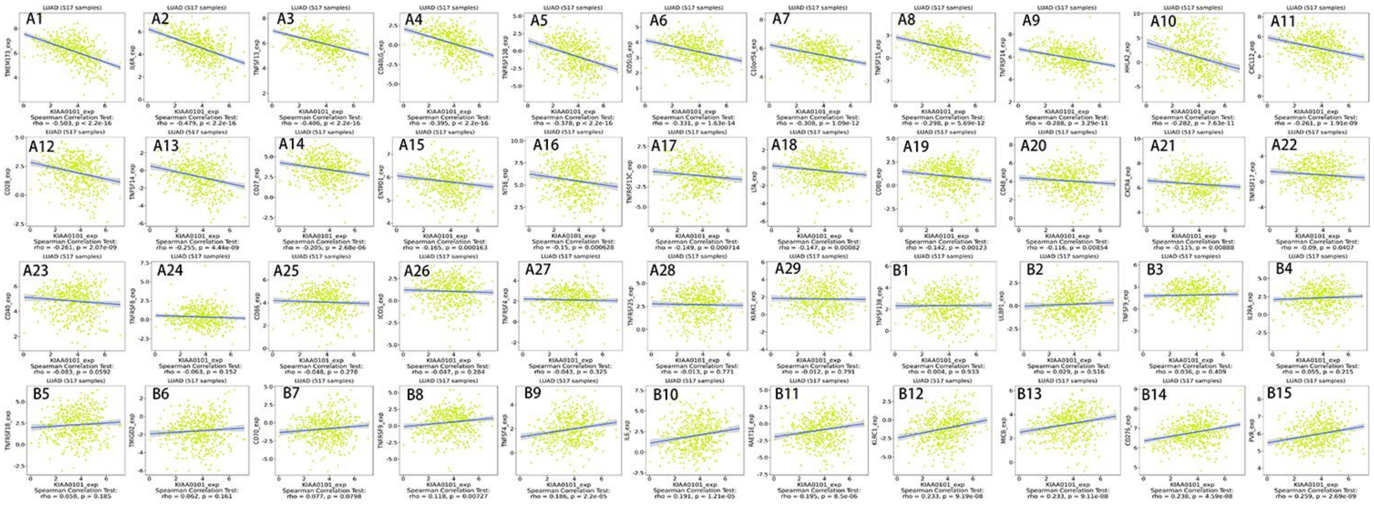

Supplementary Figure 10. The correlation between the expression of *KIAA0101* and immunostimulators. (**A1**–**A29**) are scatter plots of the negative correlations between *KIAA0101* expression and Immunostimulators in the treatment of lung adenocarcinoma. (**B1**–**B15**) are scatter plots of the positive correlations between *KIAA0101* expression and Immunostimulators in the treatment of lung adenocarcinoma.

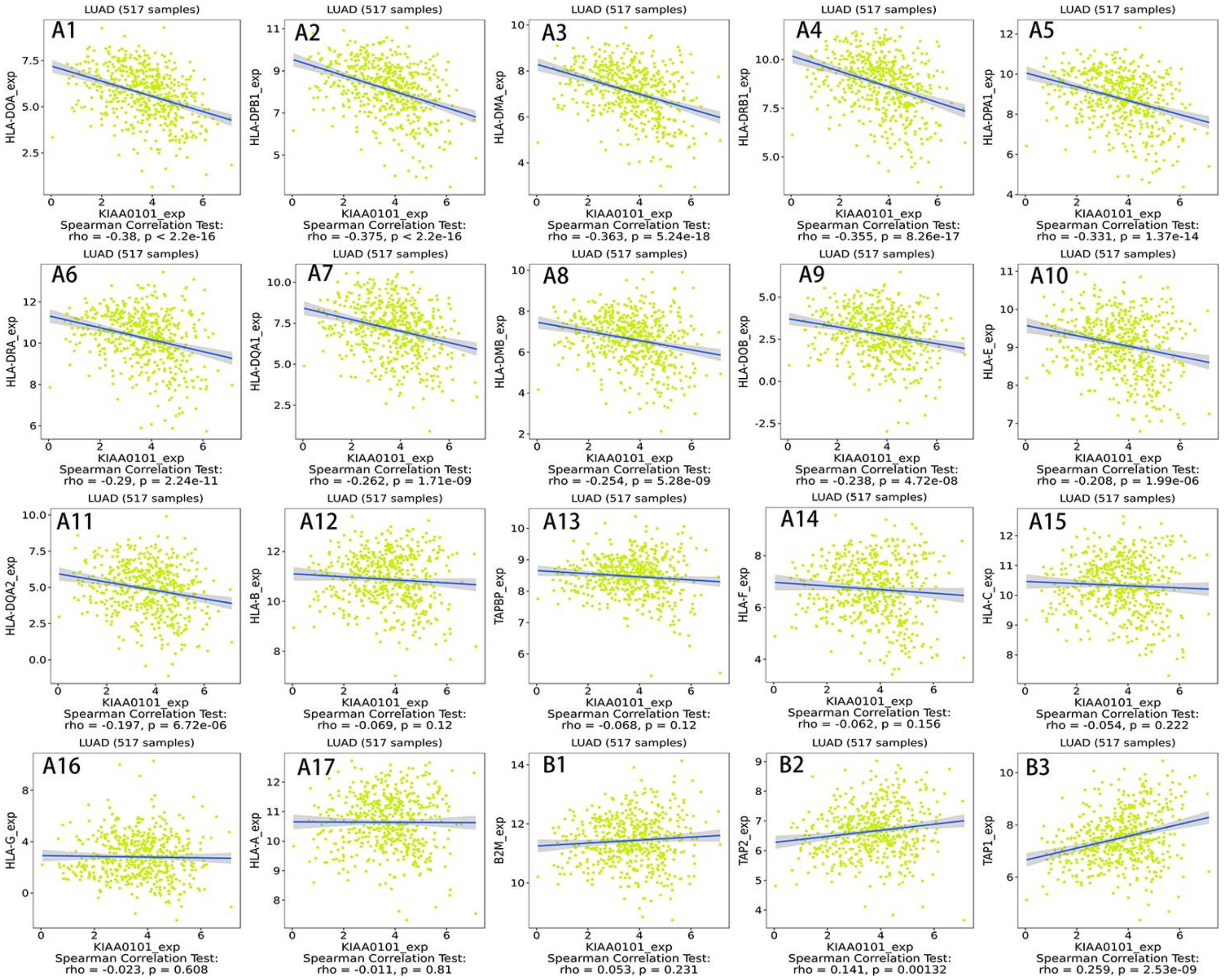

Supplementary Figure 11. The correlation between the expression of *KIAA0101* and MHC molecules. (**A1**–**A17**) are scatter plots of the negative correlations between *KIAA0101* expression and MHC molecules in the treatment of lung adenocarcinoma. (**B1**–**B3**) are scatter plots of the positive correlations between *KIAA0101* expression and MHC molecules in the treatment of lung adenocarcinoma.

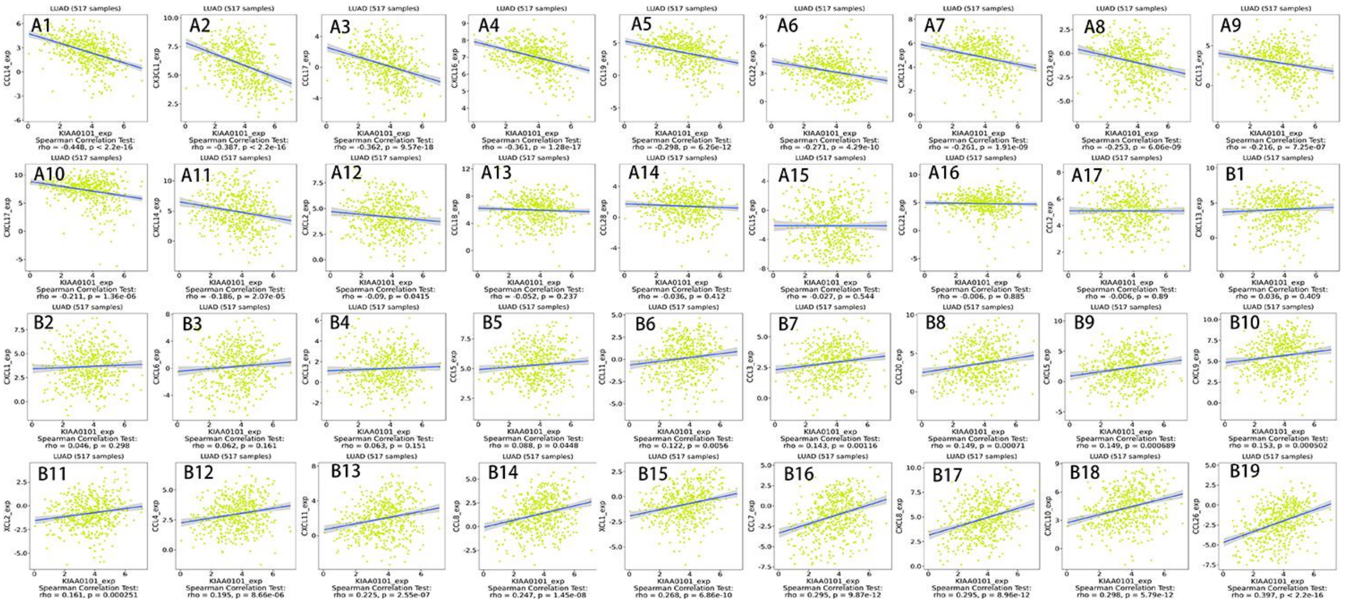

Supplementary Figure 12. The correlation between the expression of *KIAA0101* and chemokines. (**A1**–**A17**) are scatter plots of the negative correlations between *KIAA0101* expression and chemokines in the treatment of lung adenocarcinoma. (**B1**–**B19**) are scatter plots of the positive correlations between *KIAA0101* expression and chemokines in the treatment of lung adenocarcinoma.

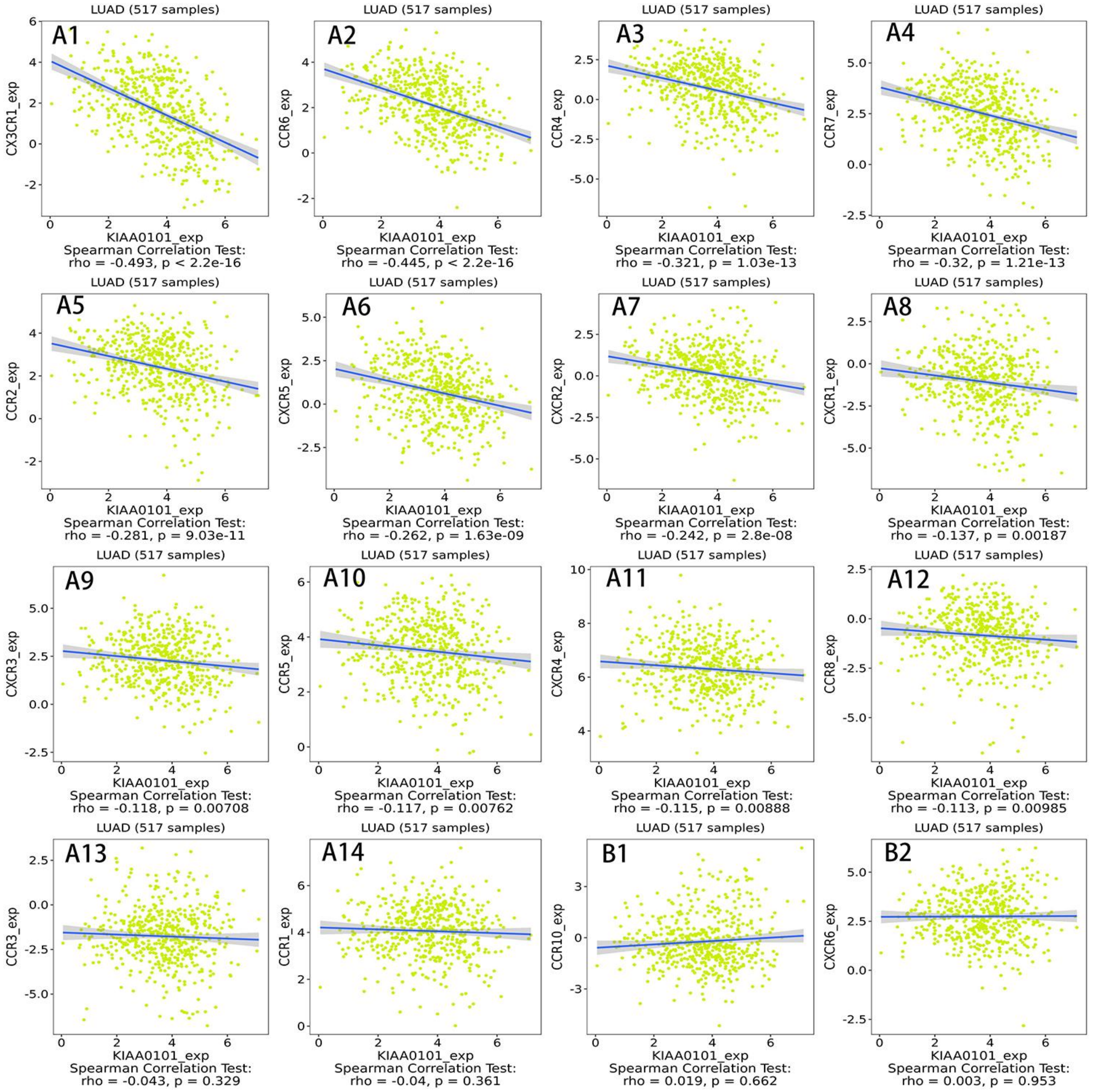

Supplementary Figure 13. The correlation between the expression of *KIAA0101* and receptors. (**A1**–**A14**) are scatter plots of the negative correlation between *KIAA0101* expression and receptors in the treatment of lung adenocarcinoma. (**B1**–**B2**) are scatter plots of the positive correlation between *KIAA0101* expression and receptors in the treatment of lung adenocarcinoma.

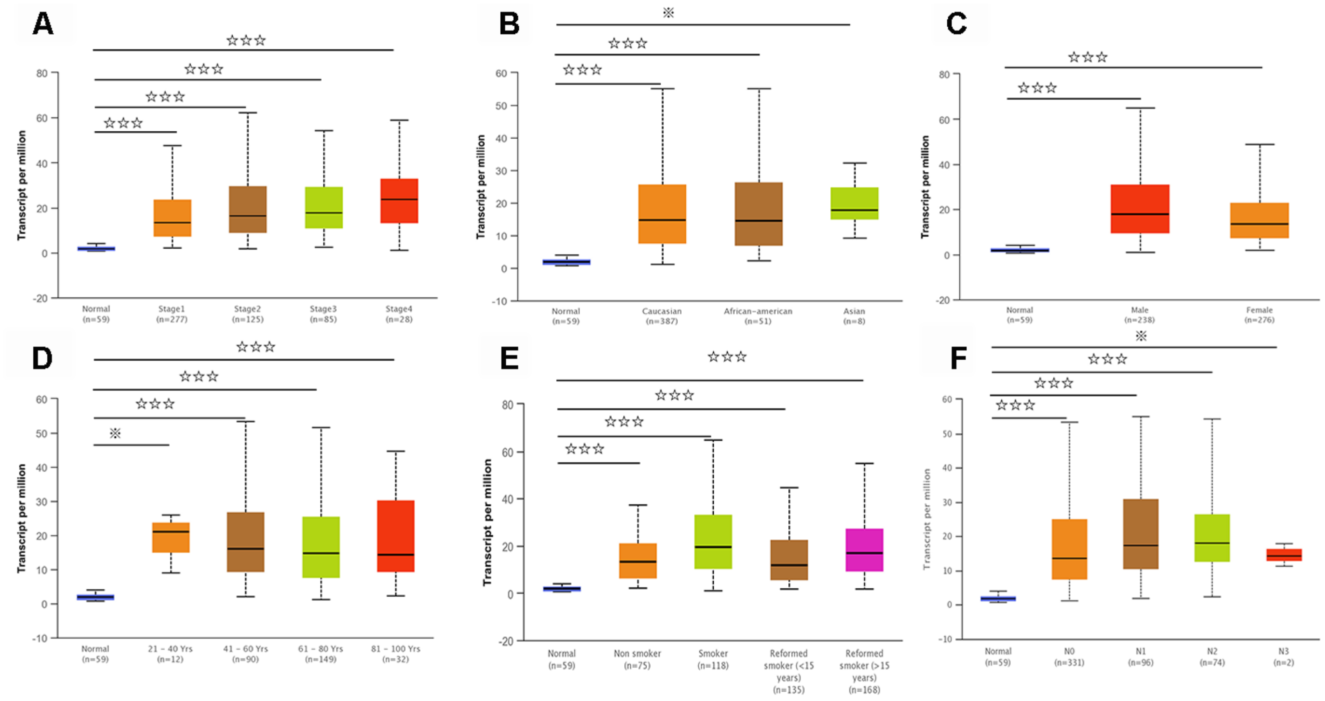

Supplementary Figure 14. ***KIAA0101*** **transcription in subgroups of patients with lung adenocarcinoma, stratified based on sex, age, and other criteria (UALCAN).** (**A**) Individual cancer stages; (**B**) patient's ethnicity; (**C**) patient's sex; (**D**) patient's age; (**E**) patient's smoking habits; (**F**) patient's metastasis status. ※ P>0.05, ☆ P < 0.05; ☆☆, P < 0.01; ☆☆☆, P < 0.001.
